# Mn(II), Fe(II), and Co(II) Aryloxides: Steric and Dispersion Effects and the Thermal Rearrangement of a Cobalt Aryloxide to a Co(II) Semiquinone Complex

**DOI:** 10.1021/acs.inorgchem.3c00610

**Published:** 2023-06-21

**Authors:** Connor P. McLoughlin, James C. Fettinger, Philip P. Power

**Affiliations:** Department of Chemistry, University of California, Davis, California 95616, United States

## Abstract

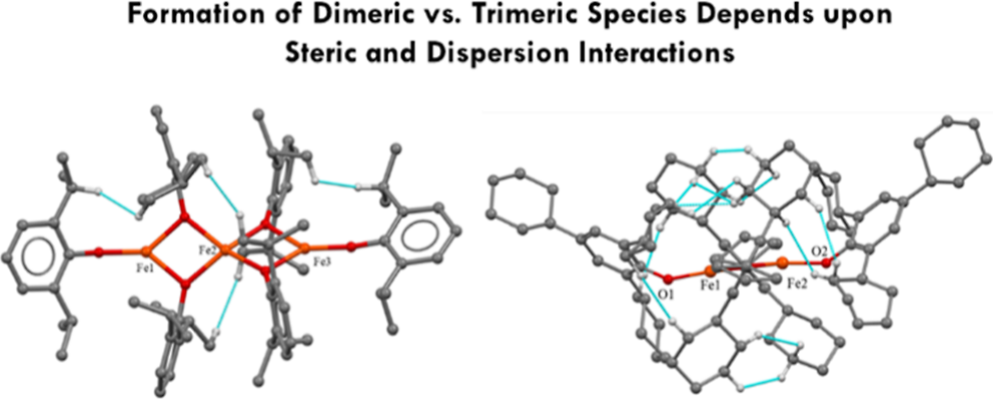

A series of Mn(II), Fe(II), and Co(II) bisaryloxide dimers ([M(OC_6_H_2_-2,4,6-Cy_3_)_2_]_2_ {M = Mn (**1**), Fe (**2**), and Co (**3**)} were synthesized by the addition of 2,4,6-tricyclohexylphenol (HOC_6_H_2_-2,4,6-Cy_3_) to the silyl amido dimers [M(N(SiMe_3_)_2_)_2_]_2_ (M = Mn, Fe, Co; Cy = cyclohexyl). An unexpected and unique Co(II) phenoxide derivative (**4**), [Co(OC_6_H_2_-2,4,6-Cy_3_)(O_2_C_6_H-3,5,6-Cy_3_)]_2_, was obtained via ligand rearrangement of **3** at ca. 180 °C. This yielded **4** in which there are two unchanged, bridging phenoxide ligands as well as a terminal bidentate semiquinone ligand bound to each cobalt. Complexes **1** and **2** did not undergo such a rearrangement under the same conditions; both are thermally stable to temperatures exceeding 250 °C and feature numerous short-contact (<2.5 Å) H···H interactions consistent with the presence of dispersion stabilization. Use of the aryloxide ligand −OC_6_H_3_-2,6-Pr^*i*^_2_ (Pr^*i*^ = isopropyl), which is sterically similar to −OC_6_H_2_-2,4,6-Cy_3_ but produces fewer close H···H interactions, gave the trimeric species [M(OC_6_H_3_-2,6-Pr^*i*^_2_)_2_]_3_ {M = Fe (**5**) or Co (**6**)} which feature a linear array of three metal atoms bridged by aryloxides. The higher association number in **5** and **6** in comparison to that of **1–3** is due to the lower dispersion energy donor properties of the −OC_6_H_3_-2,6-Pr^*i*^_2_ ligand and the lower stabilization it produces.

## Abstract 2

Six new transition-metal aryloxides were synthesized and characterized in order to compare the steric and dispersive characteristics of their substituents with those of previously available species. The studies show that the dispersive energy donor characteristics of the −C_6_H_2_-2,4,6-Cy_3_ substituent exceed those of other organic groups such as −C_6_H_3_-2,6-Pr^*i*^_2_ or −C_6_H_2_-2,4,6-Bu^*t*^_3_ and exert an important effect on their thermal stability.

## Introduction

First synthesized by Bürger and Wannagat in 1963, the transition-metal bissilylamides [M(N(SiMe_3_)_2_)_2_]_2_ (M = Mn, Co, Ni) are crucial for the development of low-coordinate (coordination numbers 2 or 3) open-shell transition-metal chemistry. These compounds are also important as convenient synthons for numerous other low-coordinate metal complexes under mild conditions.^1,2^ Their iron(II) bissilylamido congener [Fe(N(SiMe_3_)_2_)_2_]_2_, synthesized in 1988, was shown to have a similar structure and behavior to its Mn and Co analogues.^3^ However, in 1978, it emerged that the original syntheses^1,2^ of [M(N(SiMe_3_)_2_)_2_]_2_ (M = Mn, Co, Ni) by Bürger and Wannagat actually described the tetrahydrofuran complexes, M(N(SiMe_3_)_2_)_2_(THF) (M = Mn, Co), rather than the THF-free metal bissilylamides as reported originally.^4^ Similarly, Fe(N(SiMe_3_)_2_)_2_(THF) was isolated when the silylamide was prepared in THF.^5^ However, the use of diethyl ether instead of THF as a solvent afforded the THF-free complexes [M(N(SiMe_3_)_2_)_2_]_2_ (M = Mn, Fe, Co, Ni) due to the lower donor properties of Et_2_O and its greater steric demand in precluding its coordination.^3,4,6−11^ Nonetheless, it was not until 2013 that a clear distinction between the dichroic red/olive [Co(N(SiMe_3_)_2_)_2_]_2_ and the bright-green Co(N(SiMe_3_)_2_)_2_(THF) complex was recognized.^8,9^ One of the more important features of these silylamides arises from the pKa (25.8)^12^ of 1,1,1,3,3,3-hexamethyldisilazane (HMDS) which facilitates protonolysis reactions with alkyl or aryl alcohols. For example, reactions of [M(N(SiMe_3_)_2_)_2_]_2_ (M = Mn, Fe, Co) with 2,4,6-tri-*t*-butylphenol, HOC(C_6_H_11_)_3_, HOC(4-MeC_6_H_4_)_3_, or HOSiPh_3_ yielded the corresponding neutral, dimeric metal phenoxides or siloxides, while the use of trityl alcohol produced mononuclear, distorted tetrahedral metal coordination in the presence of Lewis bases.^13,14^ Further examples using boryloxides (−OBR_2_) as ligands also produced products with M_2_O_2_ core structures.^15^ Additionally, the use of adamantyl-substituted aryloxides afforded monomeric products,^16^ and substituted di-*t*-butylphenols formed dimers unless coordinated by ethereal solvents or ammonia.^17^ In parallel work, the reaction of [Co(N(SiMe_3_)_2_)_2_]_2_ with 3,5-di-*tert*-butylcatechol resulted in the formation of a tetrameric Co(II) catecholate, featuring a cubane-like Co_4_O_4_ core which was spectroscopically and structurally characterized.^18^

The importance of dispersion energies in the stabilization of unusual coordination numbers, bonding types, or oxidation states in several transition-metal species^19,20^ has been supported by dispersion-modified DFT calculations. For example, the close interligand H···H contacts in the transition-metal (IV) norbornyls, originally reported by Bower and Tennent in 1972, are key for their stability.^21−23^ Further calculations^24^ revealed that dispersion energy stabilization in these sterically crowded complexes can range from a few kcal mol^–1^ to above 35 kcal mol^–1^. Fürstner and co-workers also showed that the homoleptic iron(IV) cyclohexyl complex, “FeCy_4_”, which featured extensive H···H contacts between the four cyclohexyl groups conferred metastability to this unique species.^25^ In 2018, Schreiner and co-workers provided a cogent illustration of the importance of dispersion energies for stability when they showed that the stability of trityl radicals increases with substitution by alkyl groups due to increasing interligand dispersion interactions.^26^ Low-coordinate metal complexes were also isolated using highly sterically encumbering terphenyl ligands, but their syntheses are often laborious.^27−30^ Thus, we sought to employ a new dispersion energy donor ligand that is not as sterically encumbering as those in previously reported metal(II) aryloxides but features substituents that produce extensive interligand H···H contacts. The substituent 2,4,6-tricyclohexylphenol has numerous C–H moieties available for potential dispersion interactions. Consequently, we decided to investigate its ligand characteristics, including its dispersion effects.^24^ From a steric perspective, the 2,4,6-tricyclohexylphenoxy substituent most closely resembles the related 2,4,6-tri-isopropylphenoxy or 2,6-di-isopropylphenoxy species.^31,32^ In passing, we note that no homoleptic 2,4,6-tri-isopropylphenol or 2,6-di-isopropylphenol complexes of the metals Mn(II), Fe(II), and Co(II) have been characterized. In fact, just ca. 10 compounds of the type [M(OR)_2_]_*n*_ (M = Mn, Fe, Co, *n* = 2,3) of any kind have been structurally characterized, although a number of such complexes stabilized by σ-donor Lewis bases or solvent molecules such as ether, pyridine, ammonia, or THF are known.^13−17^ Herein, we report the synthesis and characterization of six neutral metal(II) aryloxides (M = Mn, Fe, Co) synthesized from 2,4,6-tricyclohexylphenol or 2,6-di-isopropylphenol and the respective bissilylamides [M(N(SiMe_3_)_2_)_2_]_2_ (M = Mn, Fe, Co) as synthons to demonstrate how dispersion energy donor stabilization affects physical properties and structures.

## Experimental Section

### General Considerations

All manipulations were carried out under anaerobic and anhydrous conditions by using standard Schlenk techniques or in a Vacuum Atmospheres OMNI-Lab dry box under an atmosphere of dry argon or nitrogen. Solvents were dried by the method of Grubbs^33^ and co-workers, stored over potassium or sodium, and then degassed by the freeze–pump–thaw method. All physical measurements were made under strictly anaerobic and anhydrous conditions. NMR spectra were recorded on a Varian Inova 600 MHz spectrometer or a Bruker 400 MHz AVANCE III HD Nanobay spectrometer, and the ^1^H NMR spectra were referenced to the residual solvent signals in deuterated benzene. Melting points of samples in flame-sealed capillaries were determined using a Meltemp II apparatus equipped with a partial immersion thermometer. Magnetic susceptibility data were collected at room temperature by Evans’ method^34^ using the indicated deuterated solvent and were corrected using the appropriate diamagnetic constants.^35^ IR spectra were recorded as Nujol mulls between CsI plates on a PerkinElmer 1430 spectrometer. UV–vis spectra were recorded as dilute hexane or toluene solutions in 3.5 mL quartz cuvettes using an Olis 17 modernized Cary 14 UV–vis–near-IR spectrophotometer. Unless otherwise stated, all materials were obtained from commercial sources and used as received. The ligand 2,4,6-tricyclohexylphenol is commercially available in large quantities but was donated by Toray Industries, Inc. and used without further purification. The ligand 2,6-di-isopropylphenol was purchased from Alfa Aesar and purified by distillation. The metal(II) bissilylamides [M(N(SiMe_3_)_2_)_2_]_2_ (M = Mn, Fe, Co) were prepared as described in ref (36).

#### [Mn(OC_6_H_2_-2,4,6-Cy_3_)_2_]_2_ (**1**)

[Mn(N(SiMe_3_)_2_)_2_]_2_ (0.417 g, 0.555 mmol) was added to a Schlenk tube containing 0.756 g (2.220 mmol) of 2,4,6-tricyclohexylphenol. The flask was briefly placed under dynamic vacuum, sealed, and heated with stirring to ca. 60 °C for ca. 5 min. The flask was removed from the heat source and immediately placed under dynamic vacuum for a further 5 min to remove the volatile materials. This left a green residue which was dissolved in benzene (15 mL) and placed in a ca. 8 °C refrigerator to give 0.365 g (80.2%) of **1** as pale-green rectangular blocks upon cooling for 24 h., mp > 250 °C, μ_eff_: 5.9 μ_B_ (25 °C). ^1^H NMR (600 MHz, benzene-*d*_6_): δ 7.29, 7.03, 4.33, 3.28, 3.09, 2.76, 2.11, 1.99, 1.96, 1.93, 1.91, 1.79, 1.76, 1.74, 1.68, 1.53, 1.49, 1.47, 1.43, 1.35, 1.31, 1.25, 0.89, 0.30, 0.22. UV–vis λ/nm (ε/M^–1^ cm^–1^): 234 (15,000), 281 (7700). IR (Nujol; ṽ/cm^–1^): 2930s, 2860s, 1580w, 1460s, 1450s, 1378m, 1365m, 1355m, 1300w, 1260s, 1190w, 1180w, 1150w, 1120w, 1090m, 1020s, 950w, 862w, 800s, 675w, 535w, 455w.

#### [Fe(OC_6_H_2_-2,4,6-Cy_3_)_2_]_2_ (**2**)

[Fe(N(SiMe_3_)_2_)_2_]_2_ (0.389 g, 0.517 mmol) was added to a Schlenk tube containing 0.704 g (2.068 mmol) of 2,4,6-tricyclohexylphenol. The flask was briefly placed under dynamic vacuum, then sealed, and heated to ca. 50 °C with stirring for ca. 5 min. The flask was removed from the heat source and immediately subjected to dynamic vacuum for 5 min to remove volatile materials. This resulted in a solid yellow residue which was dissolved in ca. 15 mL of benzene. Cooling in a ca. 8 °C refrigerator for 24 h gave 0.321 g (75.5%) of **2** as yellow rectangular blocks, mp > 250 °C, μ_eff_: 3.9 μ_B_ (25 °C). ^1^H NMR (600 MHz, benzene-*d*_6_): δ 78.14, 40.64, 36.85, 27.37, 18.08, 14.48, 13.27, 10.67, 9.30, 8.29, 6.08, 2.12, 1.30, 1.03, −0.15, −0.34, −2.01, −3.52, 3.82, −6.60, −8.58, −9.61, −15.33, −17.18, −22.19. UV–vis λ/nm (ε/M^–1^ cm^–1^): 282 nm (11,000), 310 nm (4100). IR (Nujol; ṽ/cm^–1^): 2930s, 2850s, 2680w, 1590w, 1445s, 1372m, 1352m, 1345m, 1290m, 1281m, 1260m, 1251m, 1200w, 1171w, 1148w, 1115w, 1035w, 952w, 860m, 807w, 753w, 726w, 690w, 655w, 525w, 460w.

#### [Co(OC_6_H_2_-2,4,6-Cy_3_)_2_]_2_ (**3**)

[Co(N(SiMe_3_)_2_)_2_]_2_ (0.799 g, 1.052 mmol) was added to a Schlenk tube along with 1.433 g (4.208 mmol) of 2,4,6-tricyclohexylphenol. The flask was cooled to 0 °C, and ca. 60 mL of hexane was added. The solution immediately assumed a dark-red color and was warmed to room temperature. The mixture was stirred for 2 h, and the solvent was removed under dynamic vacuum to yield a red residue. This was gently heated to ca. 40 °C for 15 min to remove all volatile materials. The resulting red solid was dissolved in ca. 30 mL of benzene and placed in a ca. 8 °C refrigerator. Red rectangular blocks of **3** formed from this concentrated benzene solution after 24 h to give 0.786 g (90.2%) of **3**, mp > 250 °C, μ_eff_: 5.4 μ_B_ (25 °C). ^1^H NMR (600 MHz, benzene-*d*_6_): δ 143.93, 77.54, 71.67, 36.16, 34.51, 22.97, 21.78, 19.39, 15.57, 7.12, 7.03, 6.99, 4.31, 2.73, 2.10, 1.91, 1.75, 1.67, 1.47, 1.32, 1.20, 0.30, −0.03, −1.97, −5.72, −6.86, −10.02, −10.44, −16.065, −19.78, −22.11, −50.80, −59.64. UV–vis λ/nm (ε/M^–1^ cm^–1^): 276 (7800), 499 (1500). IR (Nujol; ṽ/cm^–1^): 2940s, 2860s, 1635w, 1585m, 1460s, 1450s, 1380m, 1350m, 1300m, 1280m, 1260s, 1240m, 1200m, 1170m, 1150m, 1100s, 1020s, 950w, 890w, 865m, 800s, 730w, 695w, 670w, 540w, 465w, 390w.

#### [Co(OC_6_H_2_-2,4,6-Cy_3_)(O_2_C_6_H-3,5,6-Cy_3_)]_2_ (**4**)

[Co(N(SiMe_3_)_2_)_2_]_2_ (0.331 g, 0.436 mmol) was added to a Schlenk tube with 0.594 g (1.743 mmol) of 2,4,6-tricyclohexylphenol. The flask was placed briefly under dynamic vacuum, sealed, and heated to ca. 90 °C with stirring for 5 min. The temperature was increased to ca. 180 °C to melt the remaining unreacted material. The flask was removed from heat and immediately placed under dynamic vacuum for 5 min to remove the volatile materials, which left a red solid residue. The solid was dissolved in ca. 30 mL of hexanes and placed in an 8 °C refrigerator. A mixture of red crystals of **3** and **4** formed after 24 h to yield a total of 0.094 g of red crystals. Complex **4** was manually separated under a microscope as dark-red square crystals to give an overall yield of 0.001 g (2.14%), mp > 250 °C.

#### [Fe(OC_6_H_3_-2,6-Pr^*i*^_2_)_2_]_3_ (**5**)

[Fe(N(SiMe_3_)_2_)_2_]_2_ (0.498 g, 0.661 mmol) was added to a Schlenk tube containing 0.472 g (2.648 mmol) of 2,6-di-isopropylphenol. The flask was briefly placed under dynamic vacuum, then sealed, and heated to ca. 50 °C with stirring for ca. 5 min. The flask was removed from the heat source and immediately subjected to dynamic vacuum for ca. 10 min to remove the volatile materials. This resulted in a solid green residue which was redissolved in ca. 5 mL of benzene. The solution was filtered and cooled in a ca. 8 °C refrigerator for ca. 48 h to give 0.187 g (37.9%) of **5** as emerald-green rectangular blocks, mp > 250 °C, μ_eff_: 8.1 μ_B_ (25 °C). ^1^H NMR (400 MHz, benzene-*d*_6_): δ 97.10, 90.60, 36.49, 35.02, 30.93, 21.92, 7.04, 7.02, 4.49, 4.16, 3.35, 1.27, 1.19, 1.17, 1.02, 0.95, 0.91, 0.43, 0.25, −9.47, −11.35, −41.93, −49.41, −54.48, −73.99, −96.64. UV–vis λ/nm (ε/M^–1^ cm^–1^): 282 nm (22,550), 305 nm (10,090), 367 nm (5760). IR (Nujol; ṽ/cm^–1^): 3060m, 2950s, 2920s, 2840s, 1920w, 1880w, 1850w, 1830w, 1790w, 1690w, 1640w, 1585s, 1460s, 1375s, 1325m, 1255s, 1200m, 1170m, 1105s, 1090s, 1055m, 1040s, 955w, 930m, 900m, 880m, 830m, 800m, 790s, 745s, 710m, 680m, 600w, 560m, 465m, 400m, 285w.

#### [Co(OC_6_H_3_-2,6-Pr^*i*^_2_)_2_]_3_ (**6**)

[Co(N(SiMe_3_)_2_)_2_]_2_ (0.670 g, 0.882 mmol) was added to a Schlenk tube along with 0.629 g (3.528 mmol) of 2,6-di-isopropylphenol. The flask was placed briefly under dynamic vacuum, sealed, and heated to ca. 90 °C with stirring for 5 min. The flask was removed from the heat source and immediately subjected to dynamic vacuum for ca. 10 min to remove volatile materials. This resulted in a red solid material which was dissolved in ca. 30 mL of hot hexane and placed in a ca. 8 °C refrigerator. Ruby-red rectangular plates of **6** formed after 24 h to give 0.099 g (14.8%) of **6**, mp > 234–235 °C, μ_eff_: 7.4 μ_B_ (25 °C). ^1^H NMR (400 MHz, benzene-*d*_6_): δ 96.97, 86.14, 21.79, 4.59, 3.08, 1.89, 1.18, 0.83, 0.52, −9.45, −80.87, −85.25, −122.34. UV–vis λ/nm (ε/M^–1^ cm^–1^): 277 (12,350), 282 (14,040), 472 (3100). IR (Nujol; ṽ/cm^–1^): 3450s, 3100s, 2910, 2710w, 1910w, 1845w, 1785w, 1690w, 1650w, 1585m, 1450s, 1375s, 1360s, 1310s, 1250s, 1200m, 1180s, 1155m, 1105s, 1090s, 1055m, 1040s, 955w, 930m, 895m, 880m, 865m, 830s, 800s, 790s, 750s, 745s, 700m, 680s, 600m, 570w, 550m, 485m, 400m, 330w, 280w.

### X-ray Crystallographic Studies

Crystals of **1–4** suitable for X-ray crystallographic studies were obtained from concentrated benzene solutions of **1** and **2**, or a toluene solution of **3**, and a hexane solution of **4**, at ca. 5 °C after 24 h. Crystals of **5** and **6** suitable for X-ray crystallographic studies were obtained from concentrated benzene and hexane solutions, respectively, at ca. 8 °C after 24 h. Single crystals were removed from the Schlenk tube and immediately covered with a layer of hydrocarbon oil. Suitable crystals were selected, mounted on a nylon cryoloop, and then placed in the cold nitrogen stream of the diffractometer. Data for **1**, **2**, and **4** were collected at 190(2) K with Cu Kα_1_ radiation (λ = 1.5418 Å), and data for **3** and **6** were collected at 129(2) and 190(2) K, respectively, with Mo Kα_1_ radiation (λ = 0.71073 Å) using a Bruker D8 VENTURE dual-source diffractometer in conjunction with a CCD detector. Data for **5** were collected at 90(2) K with Mo Kα_1_ radiation (λ = 0.71073 Å) using a Bruker APEX II Mo diffractometer in conjunction with a CCD detector. The collected reflections were corrected for Lorentz and polarization effects and for absorption by using Blessing’s method as incorporated into the program SADABS.^37,38^ The structures were solved by direct methods and refined with the SHELXTL (2012, version 6.1) or SHELXTL (2013) software packages.^39^ Refinement was by full-matrix least-square procedures, with all carbon-bound hydrogen atoms included in calculated positions and treated as riding atoms. The thermal ellipsoid plots were drawn using OLEX2 software.^40^

## Results and Discussion

**Figure 1:**
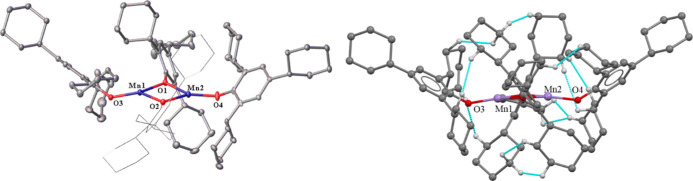
Left: crystal structure of **1** with thermal ellipsoids shown at a 30% probability. Hydrogen atoms are not shown. Important distances and angles: Mn1···Mn2 3.085(4) Å. Terminal Mn–O (avg.) 1.859(5) Å. Bridging Mn–O (avg.) 2.017(11) Å. Terminal C–O (avg.) 1.373(28) Å. Bridging C–O (avg.) 1.371(1) Å. Sum of angles at Mn1: 359.25(10). Sum of angles at Mn2: 359.64(17). R_1_: 0.054. Right: molecular model showing interligand close contacts (≤2.5 Å) in **1** depicted in blue, only hydrogen atoms participating in these close contacts are shown.

**Scheme 1:**
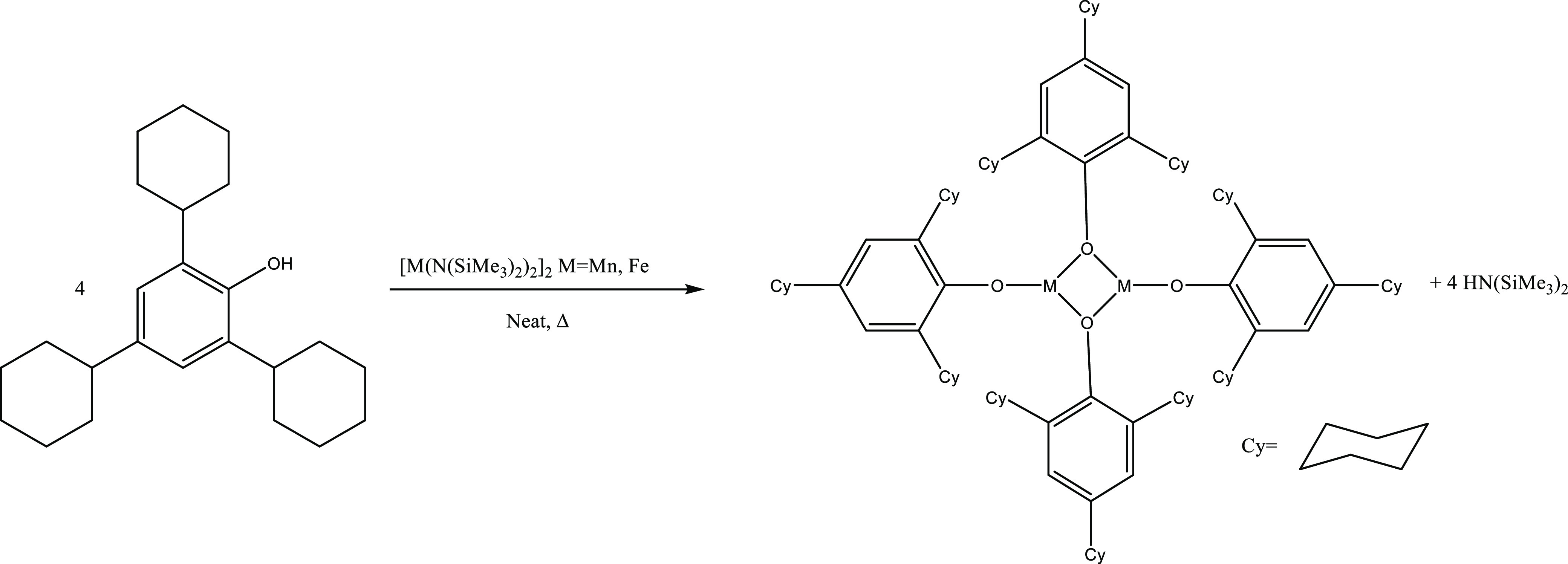
Solvent-Free Protonolysis of [M(N(SiMe_3_)_2_)_2_]_2_ (M = Mn, Fe) with 2,4,6-Tricyclohexylphenol (HOC_6_H_2_-2,4,6-Cy_3_) to Form [M(OC_6_H_2_-2,4,6-Cy_3_)_2_]_2_ [M = Mn (**1**) and Fe (**2**)]

Complex **1** was prepared by combining [Mn(N(SiMe_3_)_2_)_2_]_2_ and HOC_6_H_2_-2,4,6-Cy_3_ in a Schlenk flask and heating in an oil bath at the melting point of [Mn(N(SiMe_3_)_2_)_2_]_2_, ca. 58 °C (Scheme 1). The melting point of 2,4,6-tricyclohexylphenol is similar (ca. 50 °C),^36^ and the reaction proceeds quickly as soon as either reagent liquifies. Extraction of the resultant pale-green solid with benzene produced, upon standing, pale-green crystals of **1** that were suitable for single-crystal X-ray diffraction studies (Figure 1). The structure of **1** proved to consist of dimeric molecules with bridging aryloxide ligands and a slight pyramidalization of the three-coordinate geometry at each Mn(II) center.

The terminal and bridging Mn–O distances in **1** are longer than those in the previously reported pyridine-chelated aryloxide [Mn_2_(3,5-*t*-Bu_2_C_6_H_2_O_2_)_2_(py)_6_] (py = pyridine).^13^ The large variation in the terminal C–O distances is due to disorder at the terminal ligands from molecular librations. The Mn···Mn distance in complex **1** is shorter than that in the related dimer [Mn(OC_6_H_2_-2,4,6-Bu^*t*^_3_)_2_]_2_^13^ by ca. 0.07 Å, while the average distance for both terminals and bridging Mn–O bonds in complex **1** is ca. 0.03 Å shorter than those in [Mn(OC_6_H_2_-2,4,6-Bu^*t*^_3_)_2_]_2_ (Table 1).

**Table 1:** Selected Average Distances in **1–3** and Related Complexes

compound	M···M (Å)	terminal M–O (Å)	bridging M–O (Å)	terminal C–O (Å)	bridging C–O (Å)
**1** (Mn)	3.085(4)	1.859(5)	2.017(11)	1.373(28)	1.371(1)
[Mn(OC_6_H_2_-2,4,6-Bu^*t*^_3_)_2_]_2_^13^	3.156(2)	1.873(4)	2.050(8)	1.353(10)	1.387(9)
**2** (Fe)	2.973(9)	1.806(39)	1.957(4)	1.360(37)	1.388(1)
[Fe(OC_6_H_2_-2,4,6-Bu^*t*^_3_)_2_]_2_^13^	3.126(2)	1.822(5)	2.016(8)	1.365(18)	1.399(14)
[Fe(OC_6_H_3_-2,6-Bu^*t*^_2_)_2_]_2_^17^	3.099(12)	1.813(4)	2.020(20)	1.342(3)	1.386(2)
**3** (Co)	2.925(19)	1.795(5)	1.937(11)	1.370(5)	1.407(16)

The UV–vis spectrum in hexanes is featureless above 300 nm with two LMCT bands observable with maxima at 234 (ε = 15,000) and 281 nm (ε = 7700) due to the *d*^5^ electron configuration. The IR spectrum in Nujol shows the characteristic Mn–O bands of equal intensity at 455 and 535 cm^–1^. A magnetic moment of 5.9 μ_B_ is consistent with strong antiferromagnetic coupling between the two Mn(II) nuclei. In contrast, the spin-only value without coupling for two distinct, noninteracting *d*^5^ Mn^2+^ nuclei is calculated to be 11.84 μ_B_.^41^ Complex **1** remains unchanged up to temperatures greater than 250 °C, and there are 12 interligand close (≤2.5 Å) H···H contacts observed, presumably generating dispersion energies and stability that are comparable to those of previously reported 3-coordinate homoleptic Mn(II) dimers featuring bulkier alkyl (i.e., Bu^*t*^) groups on the central aryl ring.^13,42^

The synthesis of complex **2** was accomplished in a similar manner to that of **1** by combining the solid reagents in the Schlenk flask and placing them in an oil bath above the melting point of [Fe(N(SiMe_3_)_2_)_2_]_2_, ca. 36 °C (Scheme 1).^36^ An alternative synthetic route to **1** and **2** is via a combination of the solids in a Schlenk flask and adding hexanes (80 mL) as a solvent at ca. 0 °C.

**Figure 2:**
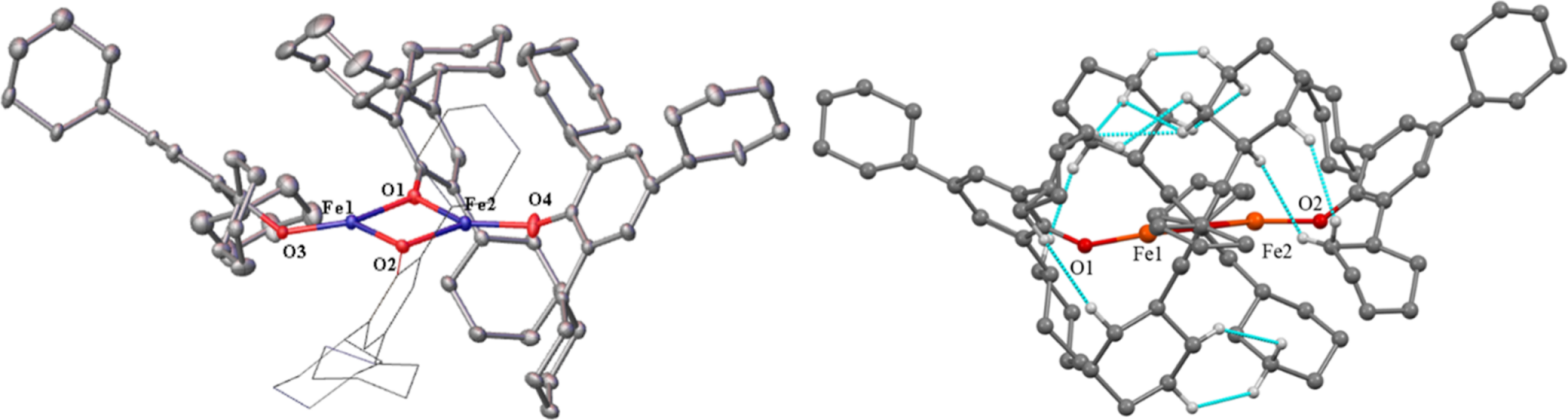
Left: crystal structure of [Fe(OC_6_H_2_-2,4,6-Cy_3_)_2_]_2_ (**2**) with thermal ellipsoids shown at 30% probability; hydrogen atoms are not shown; R_1_: 0.096. Right: molecular model showing interligand close contacts (≤2.5 Å) in **2** depicted in blue; hydrogen atoms participating in these close contacts shown in white.

When the reaction is carried out in hexanes at room temperature, the solution became dark yellow almost immediately. The ice bath was then removed, and stirring was continued for 1 h at room temperature. The solvent was pumped off along with eliminated HN(SiMe_3_)_2_ with gentle heating to ca. 35 °C. Complex **2** was then completely redissolved in hot (ca. 60 °C) benzene, and bright-yellow crystals of **2** were grown from this concentrated benzene (ca. 1.09 g in ca. 15 mL) solution in ca. 75% yield. These proved suitable for X-ray crystallographic studies (Figure 2). The structure consists of dimeric molecules with two bridging and two terminal aryloxide ligands, creating two 3-coordinate Fe(II) atoms.

The Fe_2_O_4_ core unit deviates from planarity as indicated by the interligand bond angles at each metal which do not quite sum to 360°, although the coordination is nearly planar at Fe2 (Table 2). The bridging μ^2^-O–Fe bond lengths are 0.119–0.183 Å longer than those of the terminal Fe–O bonds, with a significant range of distances reflecting the disorder in the structure. In addition, there are 12 relatively close-contact (≤2.5 Å) interactions between the hydrogens of the cyclohexyl substituents as shown in Figure 1 (right). The predicted bond length for a Fe–O single bond is 1.79 Å,^43^ and the distances observed for the terminal Fe–O bonds [av. 1.81(39) Å] match known 2-coordinate Fe(II) aryloxide monomers^13,17^ and dimers, while the bridging Fe–O bonds [av. 1.96(4) Å] are shorter than those in recently reported Fe(II) aryloxide dimers using similar ligands, such as 2,6-di-*t*-butylphenol (Table 1).^17^ The sum of the bond angles around each metal atom is similar in **1** and **2**. The Fe···Fe distance and Fe–O bonds are the shortest in iron aryloxide dimeric species (Table 2), consistent with the presence of dispersion stabilization in **1**. The distances are also consistent with the larger covalent radius of Mn (1.19 Å) in comparison to that of Fe (1.16 Å) (Table 1).^43^

**Table 2:** Selected Average Distances and Angles for **2** and Average Distances and Angles in Related Complexes

complex	Fe_1_···Fe_2_ (Å)	terminal Fe–O (Å)	bridging Fe–O (Å)	Σ angles fe1 (°)	Σ angles Fe2 (°)
[Fe(OC_6_H_2_-2,4,6-Cy_3_)_2_]_2_ (**2**)	2.973(9)	1.806(39)	1.957(4)	358.77(25)	359.84(25)
[Fe(OC_6_H_2_-2,4,6-Bu^*t*^_3_)_2_]_2_^13^	3.126(2)	1.822(5)	2.016(8)	360.00(5)	359.90(5)
[Fe(OC_6_H_3_-2,6-Bu^*t*^_2_)_2_]_2_^17^	3.099(12)	1.813(4)	2.020(20)	359.93(10)	359.98(10)
[Fe(OC_6_H_2_-2,6-Bu^*t*^_2_-4-Me)_2_]_2_^17^	3.044(5)	1.817(14)	2.004(28)	359.84(7)	359.89(7)
[Fe{N(SiMe_3_)_2_}{OC_6_H_2_-2,4,6-Bu^*t*^_3_}]_2_^13^	3.147(2)	1.905(2)	N/A	360.00(2)	360.00(2)

A qualitative indication of the dispersion energy stabilization present in **2** is evident in its high stability. The UV–vis spectrum features an LMCT band at 282 nm and a d–d transition at 310 nm. IR spectroscopy in Nujol mulls shows two bands that can be assigned to the O–Fe and μ^2^-O–Fe stretching modes at 525 and 460 cm^–1^, respectively, consistent with previously reported data for iron(II) phenoxide dimers.^13,17^ A magnetic moment of 3.9 μ_B_ was obtained via Evans’ method and indicates strong antiferromagnetic coupling between the irons in **2**, in contrast to the predicted spin-only moment of two noninteracting nuclei of 9.80 μ_B_.^41^ The magnetic moment of **1** is notably higher than that of **2**, consistent with the greater number of unpaired electrons in the Mn(II) species.

**Figure 3:**
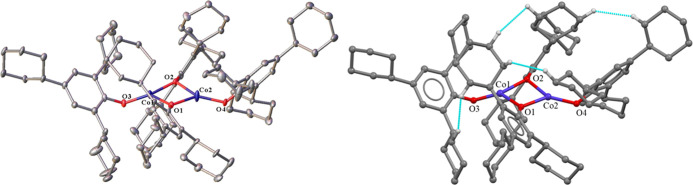
Left: crystal structure of **3** with thermal ellipsoids shown at 30% probability. Hydrogen atoms are not shown. Important distances and angles: Co1···Co2 2.925(19) Å. Terminal Co–O (avg.) 1.795(5) Å. Bridging Co–O (avg.) 1.937(11) Å. Terminal C–O (avg.) 1.370(5) Å. Bridging C–O (avg.) 1.407(16) Å. Sum of angles around Co1: 354.10(5)^°^. Sum of angles around Co2: 358.00(5). R_1_: 0.067. Right: molecular model showing interligand close contacts (≤2.5 Å) in **3** depicted in blue, hydrogen atoms participating in close contacts shown in white.

While **3** can be obtained by performing the reaction without a solvent, as in the case of **1** and **2**, pure **3** can only be isolated via the reaction of the metal bisamide with the phenol at 0 °C in hexanes (Figure 3). If the reaction is carried out with neat reagents and at a sufficiently high temperature to form a melt (>170 °C), the product is not the expected dimer **3** but a mixture of two Co(II) phenoxides, compounds **3** and **4**. Unlike its Mn(II) and Fe(II) congeners, the structure of **3** displays only 4 interligand H···H contacts either equal to, or less than, 2.5 Å after modeling the disorder surrounding the cyclohexyl substituent orientations. However, the number of contacts increases to 10 within an H···H separation of 2.6 Å. Despite the lower number of interligand H···H contacts in **3** compared to **1** and **2**, it still displays remarkable stability to temperatures greater than 250 °C.

**Figure 4:**
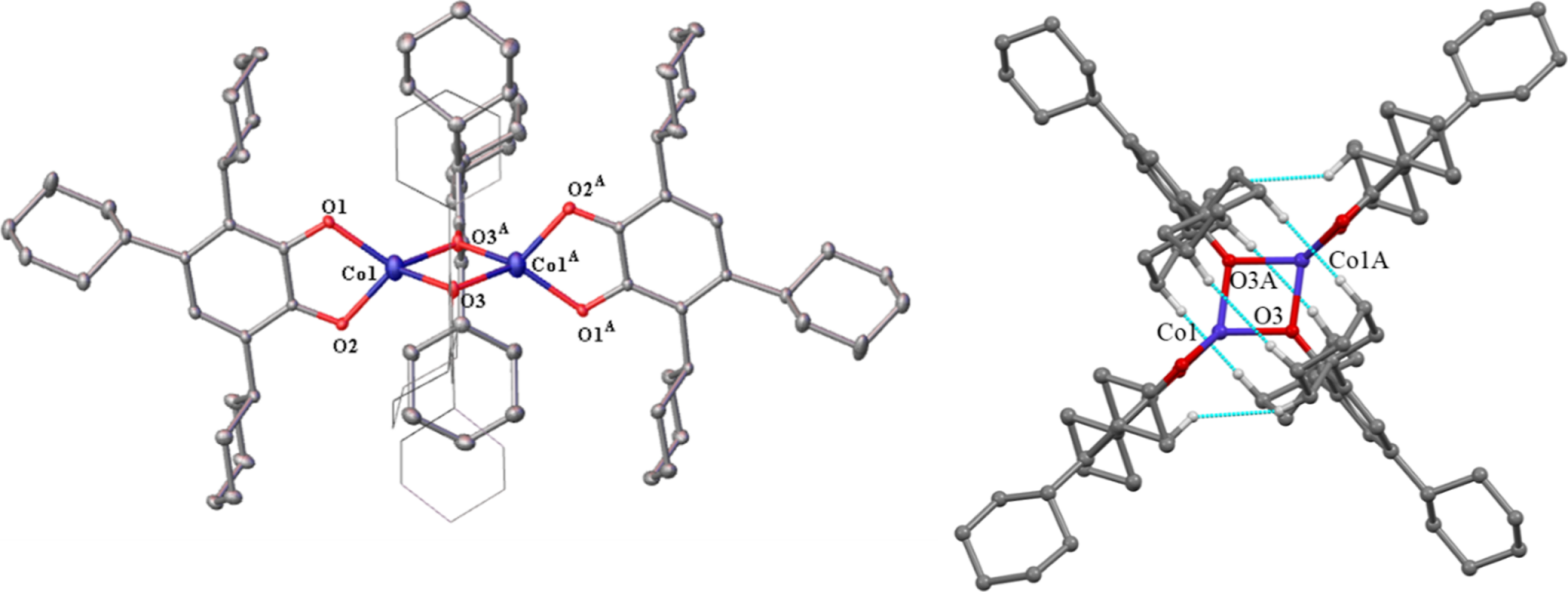
Left: crystal structure of **4** with thermal ellipsoids shown at a 30% probability, hydrogen atoms are not shown. Important distances and angles: Co1···Co1A 2.942(6) Å. Co1–O1 1.947(2) Å. Co1–O2 1.944(3) Å. Co1–O3 1.945(16) Å. Co1–O3A 1.951(16) Å. C1–C2 1.457(4) Å. O1–Co1–O2 84.59(14)°. O3–Co1–O3A 81.93(7)^°^. Co1–O3–Co1A 98.07(7)^°^. R_1_: 0.066. Right: molecular model showing interligand close contacts (≤2.5 Å) in **4** depicted in blue; hydrogen atoms participating in close contacts shown.

The structure of **4** (Figure 4) features two bidentate *o*-dioxolene ligands, each bound to a single cobalt atom, in which two additional oxygens are coordinated to two carbons in the central rings ortho to each phenolic bond, consistent with a 2,3-cyclohexyl ring shift and elimination of hydrogen from a meta position. In contrast, the bridging μ^2^-oxygen–cobalt bonds are unchanged in comparison to **3**. Both **3** and **4** are stable to temperatures beyond 250 °C. While **3** can be isolated in nearly a 90% crystalline yield from a solution-phase reaction, **4** is only obtainable via mechanical separation under a microscope in quantities sufficient to calculate a yield and determine the melting point as the colors of **3** and **4** are nearly identical and they can only be visually distinguished by their morphology. Complex **3** crystallizes as red, rectangular plates, whereas **4** crystallizes as dark-red, square blocks.

Both reactions were carried out in a 4:1 ligand-to-metal stoichiometry. While there is literature precedent^44^ for aryl α-carbon cleavage of phenolics by cobalt Schiff-base complexes to produce quinones, such reactions are typically carried out in the presence of O_2_ rather than anaerobically.^45^ Furthermore, an “alkyl-walking” mechanism was recently reported for a cobalt-catalyzed reaction in which a 1,4-shift and 1,2-shift of an alkyl group was observed via cobalt–nitrenoid insertion into alkyl-substituted arenes.^46^

To investigate if the conversion of **3** to **4** occurs as a result of oxygen contamination,^47^ pure **3** was placed under an atmosphere of dried oxygen (1 atm) in an ampoule. The solution immediately changed from dark red to brown, followed by green after 10 min. From this solution, 2,4,6-tricyclohexylphenol can be recovered and is the only organic product as evidenced by ^1^H NMR spectroscopy. Attempts to produce **4** from [Co(N(SiMe_3_)_2_)_2_]_2_ in quantitative yield using an initial 6:1 ligand-to-metal ratio were unsuccessful at all temperatures. Furthermore, **4** was only observable under a microscope in a small quantity upon reacting pure **3** with 2 additional equivalents of 2,4,6-tricyclohexylphenol by combining the solids in a Schlenk flask and heating externally under dynamic vacuum until the products melted together (>170 °C). Further investigations into the isolation of **4** in quantitative yield are currently underway.

A comparison of the bond lengths in **4** to those in **1–3** suggests that the aromaticity of the aryl rings in the bidentate ligands has been disrupted since the carbon–carbon bond lengths lie between the values of standard single (1.53 Å) and double bonds (1.32 Å) (Table 3).^48^ The bond lengths to the terminal chelating rings in **4** resemble those in Co(II) semiquinone complexes and suggest that the phenolic ligands have undergone oxidation from phenol to a semiquinone, with preservation of the oxidation state of each cobalt atom as Co(II). Modeling of **4** to lower final residual values from ca. 8.4 to 6.6% gives bond distances that lie between the idealized and predisorder models. Nonetheless, the bond lengths in the idealized structure and predisorder model maintain distances that resemble those in Co(II) semiquinones. Nonbridging carbon–oxygen distances for catecholate complexes fall in the range of 1.35–1.37 Å while those of the semiquinones are shorter, in the range of 1.28–1.31 Å.^18,49−51^ Two dioxolene ligands bearing isopropyl and cyclohexyl groups were used to characterize Co(II) and Co(III) semiquinone complexes sharing structurally similar parameters in the chelate rings to **4** (cf. Table 3).^52^ The eclipsing of the cyclohexyl rings of the ligands in **4** (cf. Figure 4) further demonstrates the importance of dispersion forces in the stability of the complex.

**Table 3:** Comparison of Selected Chelate Ring Bond Lengths (Average) in **4** to Those of Published Co(II)/Co(III) Semiquinone (SQ) and Catecholate (Cat) Structures^a^

Complex	M–O bonds (Å)	C–O bonds (Å)	C–C (Å)	type
[Co_4_(DBCat)_4_(THF)_5.5_]^18^	1.92(14) (term.)	1.34(2) (term.)	1.37(2)	Cat
	2.11(11)	1.41(2)		
	(bridging)	(bridging)		
Co_4_(3,5-DBSQ)_8_^49^	2.05(4)	1.28(7)	1.45(9)	SQ
Co(3,5-DBCat) (3,5-DBSQ)-(bipy)^50^	1.90(6)	1.30(9)	1.45(11)	SQ
Co(3,5-DBCat) (3,5-DBSQ)-(bipy)^50^	1.87(6)	1.36(10)	1.38(12)	Cat
Co(bpy) (C_6_H_2_-3,6-di^i^Pr)_2_(THF)^52^	1.92(18)	1.29(3)	1.45(4)	SQ
Co(bpy) (C_6_H_2_-3,6-diCy)_2_(THF)^52^	2.07(21)	1.29(24)	1.45(23)	SQ
[Co(OC_6_H_2_-2,4,6-Cy_3_) (O_2_C_6_H-2,4,6-Cy_3_)]_2_ (**4**)	1.95(2)	1.29(3)	1.46(4)	SQ
reported range of catecholate distances^49^		1.35–1.37	1.37–1.41	Cat
reported range of semiquinone distances^49^		1.28–1.31	1.43–1.45	SQ

a DBCat: di-*tert*-butylcatecholate; DBSQ: di-*tert*-butylsemiquinone.

**Figure 5:**
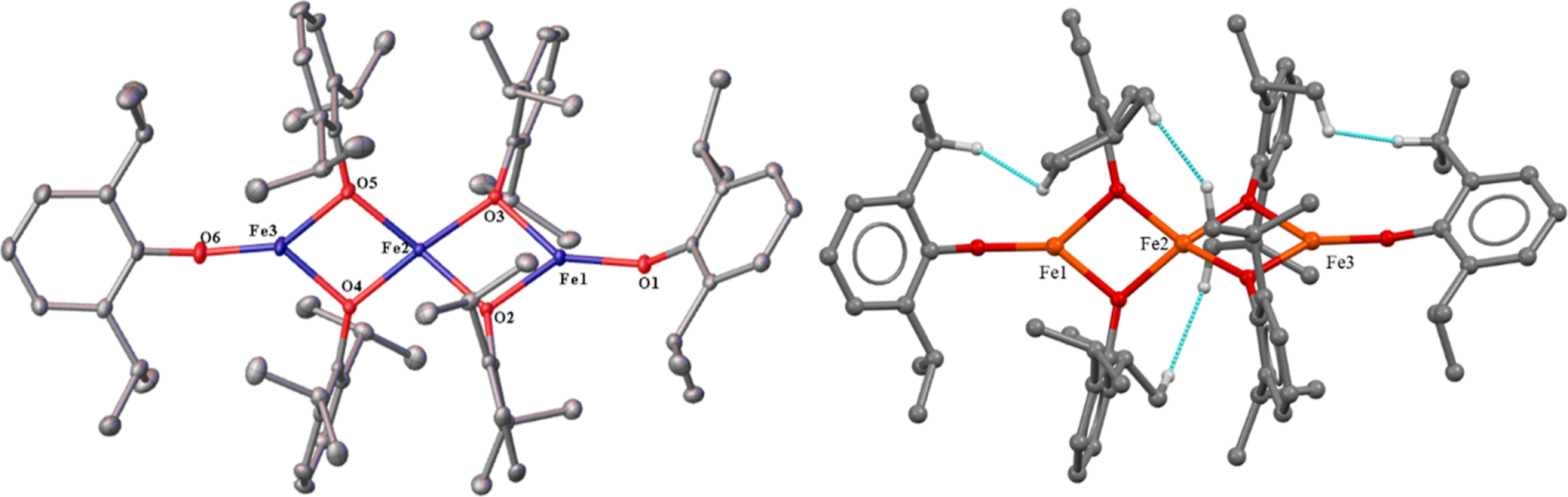
Left: crystal structure of **5** with thermal ellipsoids shown at a 30% probability; hydrogen atoms are not shown, with one of the two crystallographically distinct molecules shown. Important distances and angles: Average Fe···Fe 2.989(9) Å. Terminal Fe–O (ave.) 1.795(16) Å. Bridging Fe–O (ave.) 1.970(19) Å. Terminal C–O (ave.) 1.353(7) Å. Bridging C–O (ave.) 1.391(2) Å. O(2)–Fe(1)–O(3) 82.17(6)^°^. O(3)–Fe(2)–O(2) 80.22(6)^°^. O(4)–Fe(2)–O(5) 80.54(6)^°^. O(5)–Fe(3)–O(4) 81.99(6)°. R_1_: 0.047. Right: molecular model showing interligand close contacts (≤2.5 Å) in **5** depicted in blue, hydrogen atoms participating in close contacts shown.

No homoleptic transition-metal derivatives of the sterically similar and commercially available ligand HOC_6_H_3_-2,6-Pr^*i*^_2_ have been reported. The iron and cobalt −OC_6_H_3_-2,6-Pr^*i*^_2_ complexes **5** and **6** were synthesized similarly to **1** and **2** by conducting the reaction neatly at the melting point of the metal bissilylamides (Scheme 1). These reactions proceed rapidly in comparison to those of **1** and **2** since 2,6-di-isopropylphenol is a colorless liquid at room temperature that begins solubilizing the metal bissilylamide upon the combination of the reactants in the flask. In comparison, the combination of 2,4,6-tricyclohexylphenol with the metal bissilylamides to give **1–3** requires the formation of a melt before any reactivity is observed. Complex **5** (Figure 5) is an iron(II) aryloxide trimer that crystallizes as large emerald green blocks from benzene at ca. 8 °C. The trimeric structure is similar to that of [Mn(Mes)_2_]_3_ (Mes = Mesityl), while the use of Trip (−C_6_H_2_–2,4,6-Pr^*i*^_3_) gives the dimer [Mn(Trip)_2_].^53,54^ Complex **5** crystallizes with two crystallographically distinct molecules per asymmetric unit that includes multiple solvent molecules. The ^1^H NMR spectrum (400 MHz, C_6_D_6_, 25 °C) is consistent with a paramagnetic complex with resonances appearing between +100 and −100 ppm. A magnetic moment of 8.1 μ_B_ measured via Evans’ method^34^ demonstrates strong antiferromagnetic coupling between the three Fe^2+^ ions in the linear Fe_3_ array. The UV–vis spectrum shows three absorbances at 282 nm (22,550), 305 nm (10,000), and 367 nm (5800), while the IR spectrum shares similar features to **2** with the O–Fe and μ^2^-O–Fe stretching modes at 560 and 465 cm^–1^, respectively. Complex **5** does not melt or decay up to temperatures greater than 250 °C, with the emerald, green crystals maintaining their vibrant hue up to this temperature.

The average Fe···Fe distance in [Fe(OC_6_H_3_-2,6-Pr^*i*^_2_)_2_]_3_ (**5**) is slightly longer (by ca. 0.02 Å) than that observed in [Fe(OC_6_H_2_-2,4,6-Cy_3_)_2_]_2_ (**2**), but the average terminal Fe–O distances are similar (1.795 Å in **5** vs 1.806 Å in **2**). This is presumably due to a combination of the reduced dispersion energy donor capability of −OC_6_H_3_-2,6-Pr^*i*^_2_ in comparison to −OC_6_H_2_-2,4,6-Cy_3_ as well as the presence of a 4-coordinate Fe(II) atom in the center of the linear array. The bridging Fe–O distances around Fe2 are substantially longer than those of the terminal Fe atoms in the linear array, with an average Fe2–O distance of 1.987(8) Å vs an average distance of 1.953(3) Å to Fe1 and Fe3, with the latter distance mirroring those of complex **2** [1.957(4) Å]. The lengthening of the Fe2–O distances in comparison to Fe1 and Fe3 is expected as they are all bridging μ^2^-O–Fe2 bonds. The terminal C–O bonds in **5** [1.353(7) Å] are shorter than those of **2** [1.360(37) Å], while the bridging C–O distances in **5** are nearly identical.

**Figure 6:**
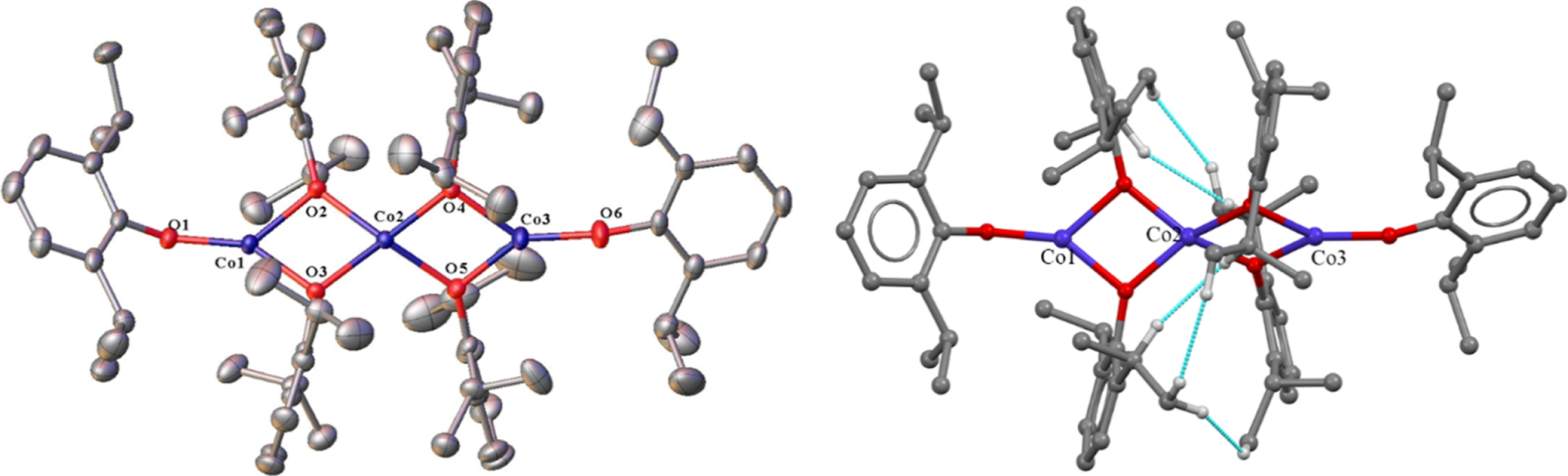
Left: crystal structure of **6** with thermal ellipsoids shown at a 30% probability; hydrogen atoms are not shown. Important distances and angles: Average Co···Co 2.970(3) Å. Terminal Co–O (ave.) 1.769(15) Å. Bridging Co–O (ave.) 1.945(16) Å. Terminal C–O (ave.) 1.346(8) Å. Bridging C–O (ave.) 1.384(1) Å. O(3)–Co(1)–O(2) 80.68(5)°. O(2)–Co(2)–O(3) 79.67(5)°. O(4)–Co(2)–O(5) 79.55(5)^°^. O(5)–Co(3)–O(4) 81.36(5)^°^. R_1_: 0.036. Right: molecular drawing showing interligand close contacts (≤2.5 Å) in **6** depicted in blue; hydrogen atoms participating in close contacts shown.

In contrast to **5**, complex **6** crystallizes from hexane with one trimer and no solvent molecules per asymmetric unit. Complex **6** (Figure 6) is structurally analogous to **5** and features a trimeric Co(II) array with six aryloxide ligands in the periphery. The ^1^H NMR spectrum (400 MHz, C_6_D_6_, 25 °C) is similar to that of **5** and features a broader range of resonances, stretching from −125 to +100 ppm. Complex **6** shows three absorbances in the UV–vis spectrum at 277 nm (12,350), 282 nm (14,040), and 472 nm (3100); however, two are below 300 nm in contrast to the iron analogue. While **5** is stable to 250 °C, [Co(OC_6_H_3_-2,6-Pr^*i*^_2_)_2_]_3_ melts from 234–235 °C but does not decompose.

The average Co···Co distance is longer in **6** than that in complex **3** by approximately 0.05 Å, while the terminal Co–O distances are shorter by nearly 0.03 Å. While the average bridging Co–O distances are longer in [Co(OC_6_H_3_-2,6-Pr^*i*^_2_)_2_]_3_ (**6**) than [Co(OC_6_H_2_-2,4,6-Cy_3_)_2_]_2_ (**3**) as a whole, the bridging Co–O distances at each terminal cobalt atom (Co1 and Co3) are shorter in **6** [1.930(5) Å) than **3** (1.937(11) Å], while the central Co2–O distances are significantly longer, averaging 1.959(8) Å. This lengthening is analogous to that of **5** as each Co–O bond is a bridging μ^2^-O–Co2 interaction that forms a 4-coordinate Co atom between two terminal 3-coordinate Co atoms. The internal O–Co–O angles are similar in **3** and **6** owing to the sterically similar isopropyl and cyclohexyl groups on the aryl ring, with those of **6** being slightly more acute in every instance but one by less than a degree.

While the similarity in the bond angles and distances in **5** and **6** to the 2,4,6-tricyclohexylaryloxo congeners demonstrates the structural similarities between 2,6-di-isopropylphenol and 2,4,6-tricyclohexylphenol, disparities arise when comparing their interligand H···H contacts within 2.5 Å and their overall structure. Complex **5** features only 4 H···H close contacts, while its 2,4,6-tricyclohexylphenoxy analogue features 12. While an analysis of H···H close contacts in complex **3** only returns 4 results ≤2.5 Å because of modeling the disorder around the ligands, the number of close contacts increases to 10 within 2.6 Å. In comparison, complex **6** features 5 within or equal to 2.5 Å and 8 within 2.6 Å. We posit that as the isopropyl groups have less −CH_2_ moieties that can participate in H···H close-contact interactions, the formation of trimeric rather than dimeric structures is observed, demonstrating the importance of dispersion energy interactions for the formation of the dimeric complexes **1–3**.

## Conclusions

Three examples of homoleptic Mn(II), Fe(II), and Co(II) aryloxide dimers were synthesized using 2,4,6-tricyclohexylphenolate as a dispersion energy donor ligand. An additional Co(II) complex, **4**, was isolated as a result of an alkyl-walking shift on the central aryl ring which formed a bidentate chelate ring at the terminal positions with unchanged bridging ligands. It is highly probable that dispersion stabilization energies from interligand H···H contacts contribute to the observed high stability of complexes **1–4**. These stabilities exceed those of previously reported Mn(II), Fe(II), and Co(II) aryloxides such as the *t*-Bu-substituted −OC_6_H_2_-2,4,6-Bu^*t*^_3_ or −OC_6_H_3_-2,6-Bu^*t*^_2_ complexes^13,17^ despite the decrease in steric bulk provided by the cyclohexyl flanking rings. A separate, novel Co(II) semiquinone **4** was isolated and characterized by X-ray crystallography; the mechanism for the formation and quantitative synthesis of **4** will require a separate study and is being actively investigated. To demonstrate the importance of H···H close-contact interactions that generate dispersion stabilization energies to complexes **1–3**, two additional complexes (**5** and **6**) were synthesized using the sterically similar 2,6-di-isopropylphenoxide ligand. [Fe(OC_6_H_3_-2,6-Pr^*i*^_2_)_2_]_3_ (**5**) and [Co(OC_6_H_3_-2,6-Pr^*i*^_2_)_2_]_3_ (**6**) are trimeric rather than dimeric and feature a linear array of metal atoms with six aryloxo ligands in the periphery, demonstrating the importance of dispersion energy stabilization in the isolation of the dimeric complexes **1–3**.

## Supporting Information Available

^1^H NMR, IR, and UV–vis spectra for **1–3** and **5–6** and crystallographic data for **1–6** (PDF)
