# Supplementary material for: Mn(II), Fe(II), and Co(II) Aryloxides: Steric and Dispersion Effects and the Thermal Rearrangement of a Cobalt Aryloxide to a Co(II) Semiquinone Complex
Source: Inorg Chem. 2023 Jun 21;62(26):10131–40. doi: 10.1021/acs.inorgchem.3c00610 (PMC10324311; doi:10.1021/acs.inorgchem.3c00610)
Supplement: Supplementary file 1 — ic3c00610_si_001.pdf [file ic3c00610_si_001.pdf]

Mn(II), Fe(II), and Co(II) Aryloxides: Steric and Dispersion Effects and the Thermal Rearrangement of a Cobalt Aryloxide to a Co(II) Semiquinone Complex

Connor P. McLoughlin, James C. Fettinger, and Philip P. Power*

Department of Chemistry, University of California, One Shields Avenue, Davis, California 95616, United States

Philip P. Power: pppower@ucdavis.edu

Supporting Information

Table of Contents:

1. Characterization data for complex **1** …………………………………………………...S3

Figure S1. ^{1}H NMR spectrum of **1** …………………………………………………...S3

Figure S2. Infrared spectrum of **1** …………………………………………………….S3

Figure S3. UV-Vis spectrum (51 µM) of **1**...……………………………………………S4

2. Characterization data for complex **2**……………………………………………………S4

Figure S4. ^{1}H NMR spectrum of **2** …………………………………………………...S4

Figure S5. Infrared spectrum of **2** …………………………………………………….S5

Figure S6. UV-Vis spectrum (51 µM) of **2**...……………………………………………S5

Figure S7. UV-Vis spectrum (710 µM) of **2**…......……………………………………………S6

3. Characterization data for complex **3** …………………………………………………………...S6

Figure S8. ^{1}H NMR spectrum of **3** …………………………………………………………...S6

Figure S9. Infrared spectrum of **3** ……………………………………………………………….S7

Figure S10. UV-Vis spectrum of **3**...……………………………………………………………….S7

4. X-Ray Crystallographic data for **1-4**…………………………………………………………...S7

Table S1. Crystal data and structure refinement for **1-4**……………………………….……...S7

5. Characterization Data for complex **5** ………………………………………………………… S9

Figure S11. ^{1}H NMR spectrum of **5** ……………………………..…………………………...S9

Figure S12. Infrared spectrum of **5**…………………………………………………………...S9

Figure S13. UV-Vis spectrum (89 μM) of **5**……………………………………………………...S10

Figure S14. UV-Vis spectrum (45 μM) of **5**………………………………………………...…....S10

6. Characterization Data for complex **6**…………………………………………………..…. S11

Figure S15. ^{1}H NMR spectrum of **6**……………..……………………………………..…. S11

Figure S16. Infrared spectrum of **6**……………..……………….……………….…………..…. S11

Figure S17. UV-Vis spectrum (27 μM) of **6**……………………………………………………...S12

Table S2. Crystal data and structure refinement for **5-6**……………………………………..S12

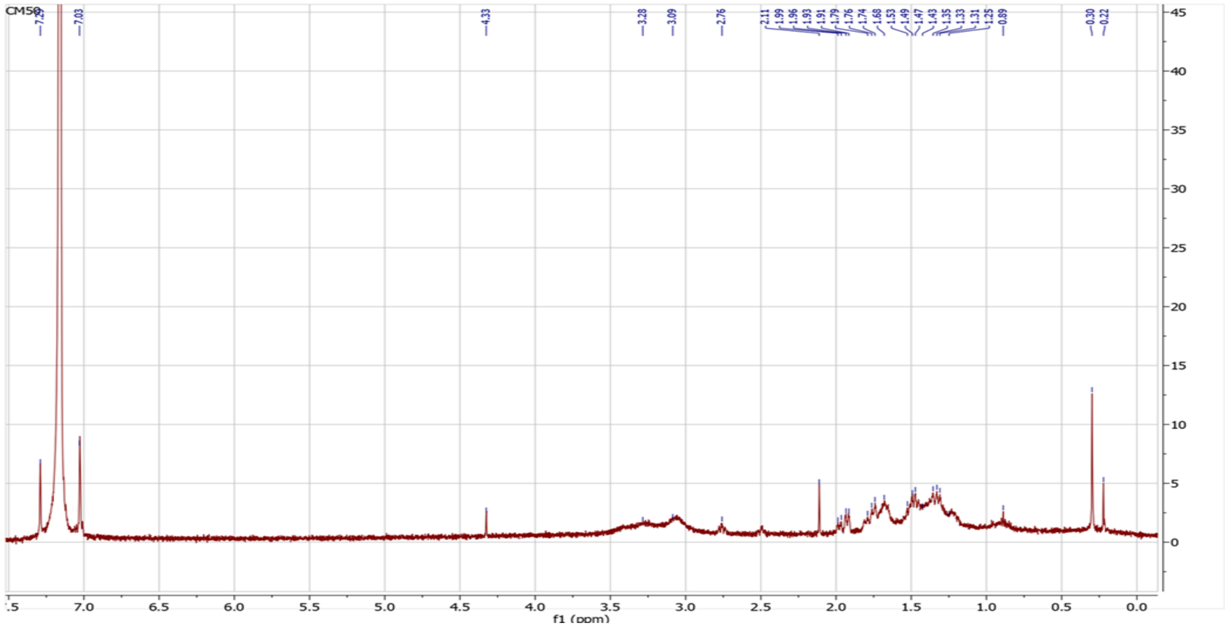

Figure S1. ^{1}H-NMR spectrum (C_6D_6, 600 MHz, 25 ^oC) of $[Mn(OC_6H_2\text{-}2,4,6\text{-}Cy_3)_2]_2$ (**1**).

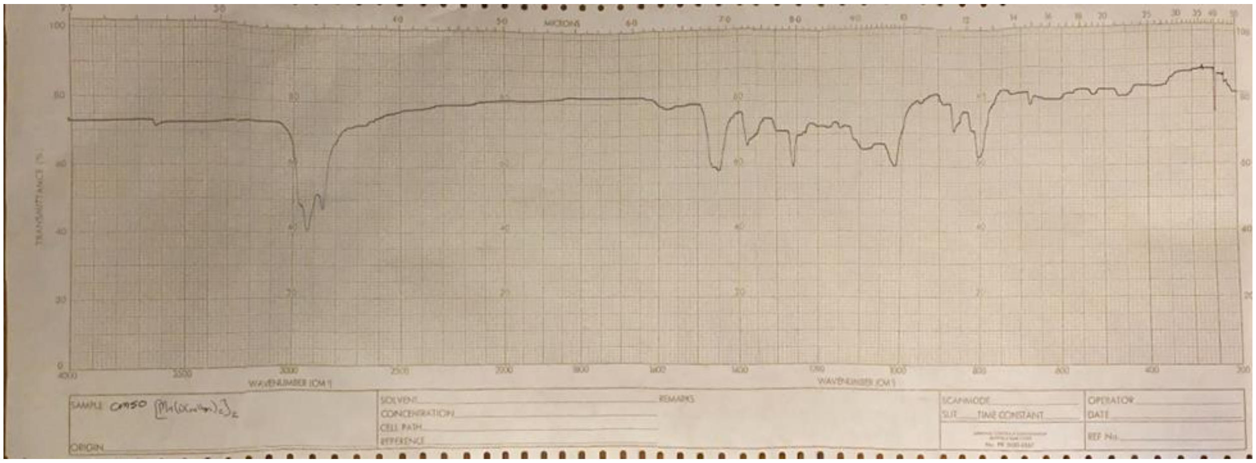

Figure S2. IR spectrum (Nujol) of $[Mn(OC_6H_2\text{-}2,4,6\text{-}Cy_3)_2]_2$ (**1**).

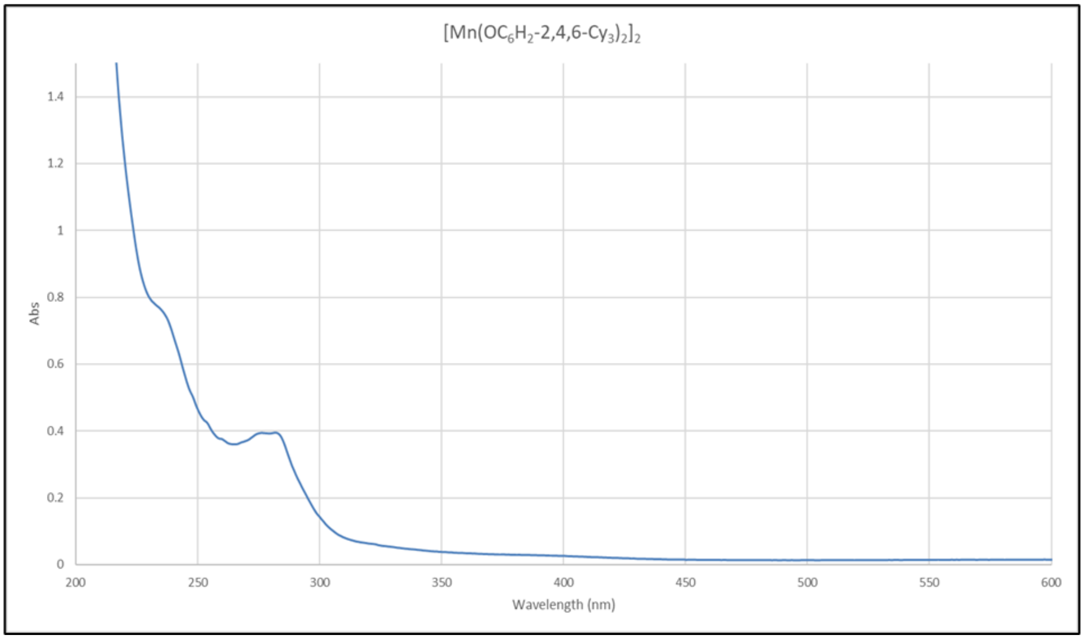

Figure S3. UV-Vis spectrum (51 µM, Hexanes) of $[Mn(OC_6H_2\text{-}2,4,6\text{-}Cy_3)_2]_2$ (**1**).

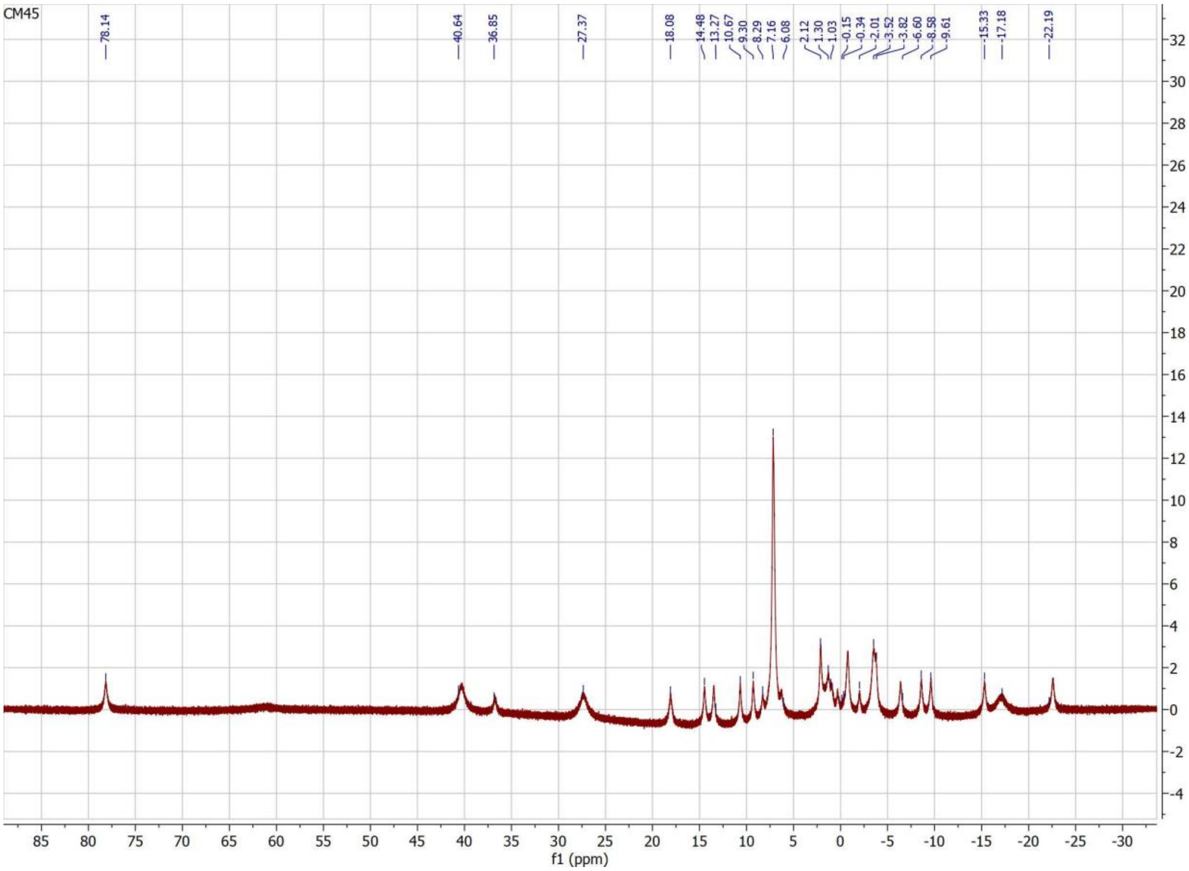

Figure S4. ^{1}H-NMR spectrum (C_6D_6, 600 MHz, 25 ^oC) of $[Fe(OC_6H_2\text{-}2,4,6\text{-}Cy_3)_2]_2$ (**2**).

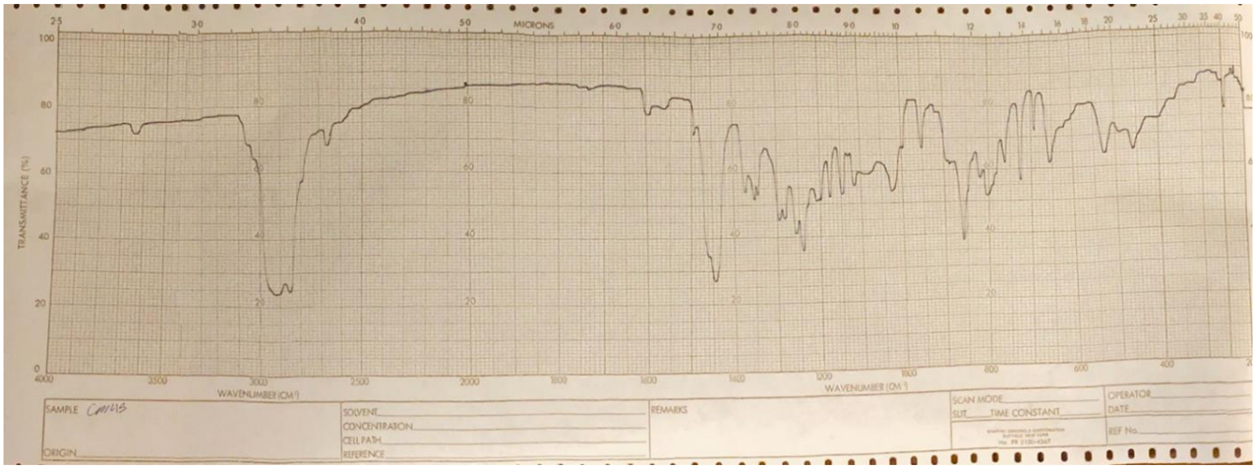

Figure S5. IR spectrum (Nujol) of [Fe(OC$_6$H$_2$-2,4,6-Cy$_3$)$_2$]$_2$ (**2**).

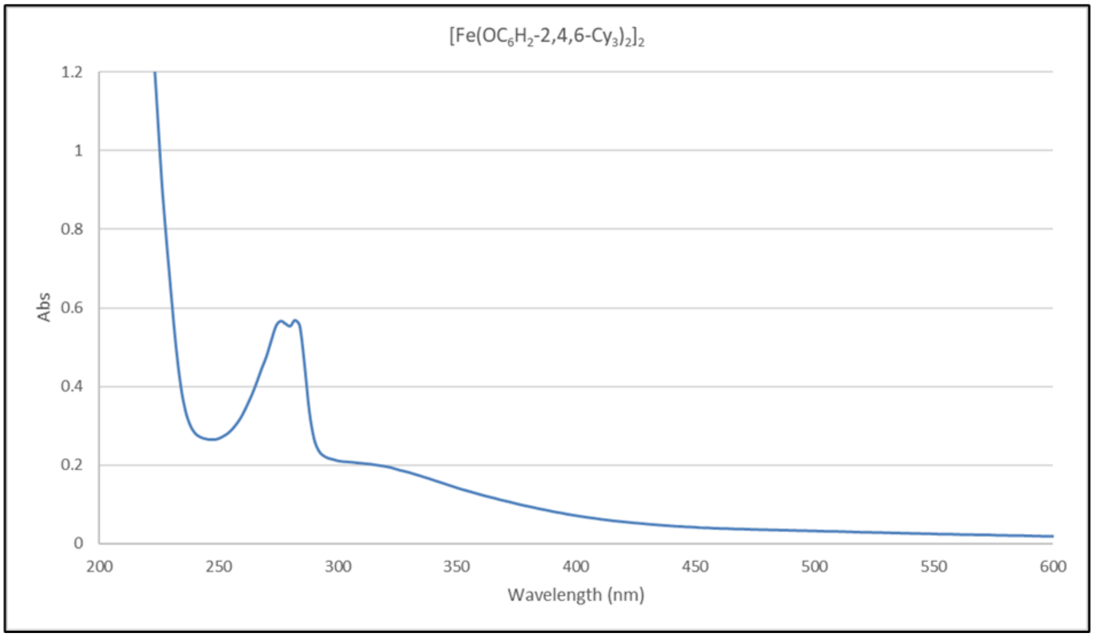

Figure S6. UV-Vis spectrum (51 µM, hexanes) of [Fe(OC$_6$H$_2$-2,4,6-Cy$_3$)$_2$]$_2$ (**2**).

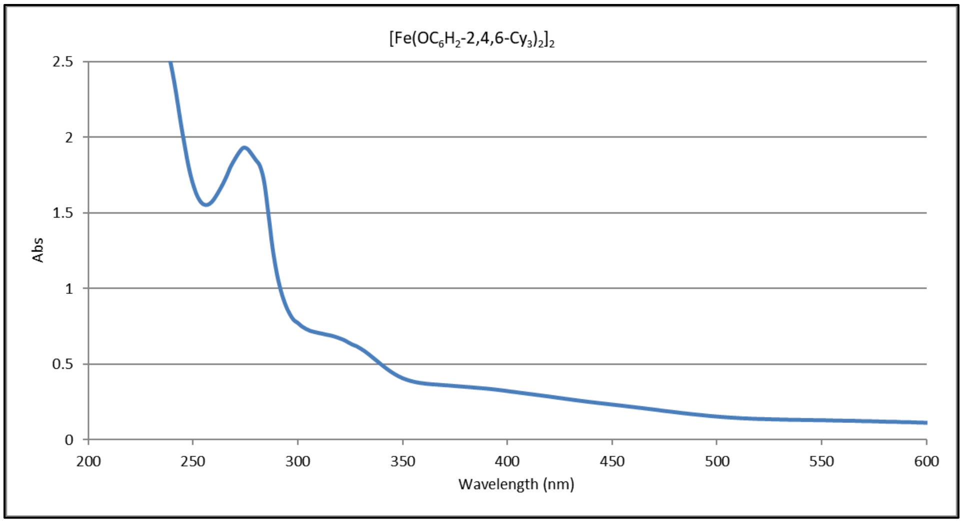

Figure S7. UV-Vis spectrum (710 µM, Hexanes) of $[Fe(OC_6H_2\text{-}2,4,6\text{-}Cy_3)_2]_2$ (**2**).

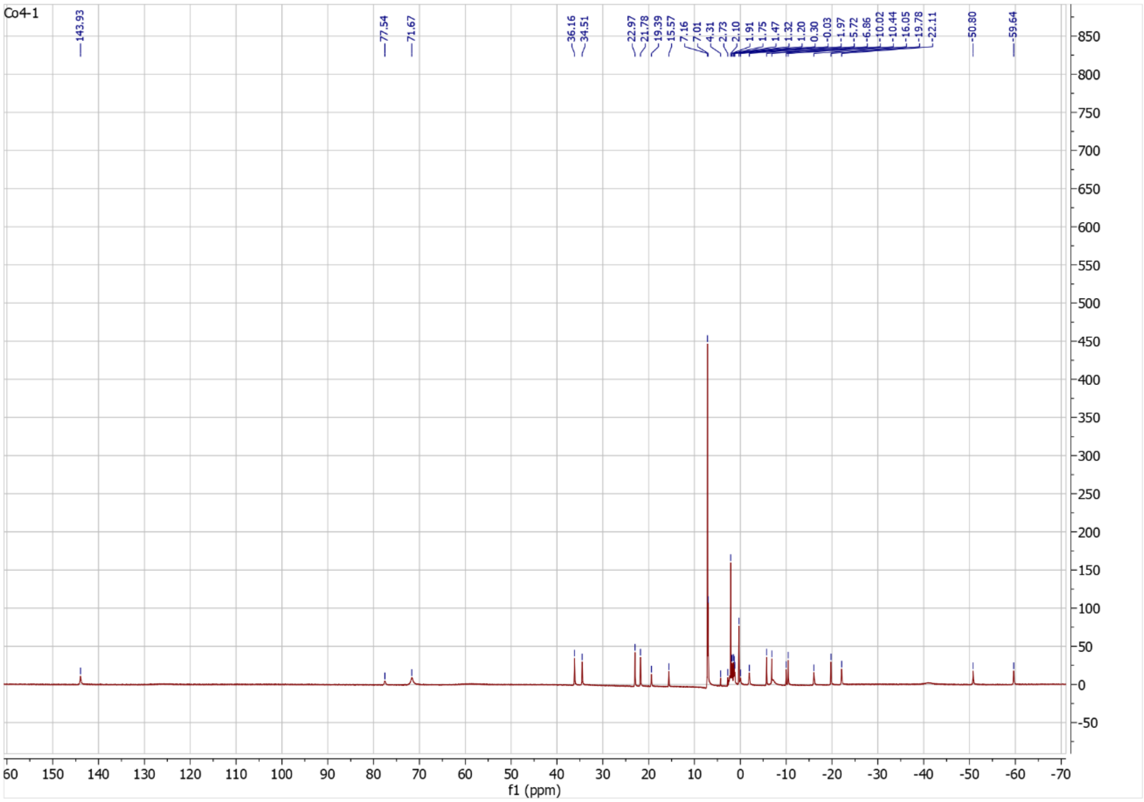

Figure S8. ^{1}H-NMR spectrum (C_6D_6, 600 MHz, 25 °C) of $[Co(OC_6H_2\text{-}2,4,6\text{-}Cy_3)_2]_2$ (**3**).

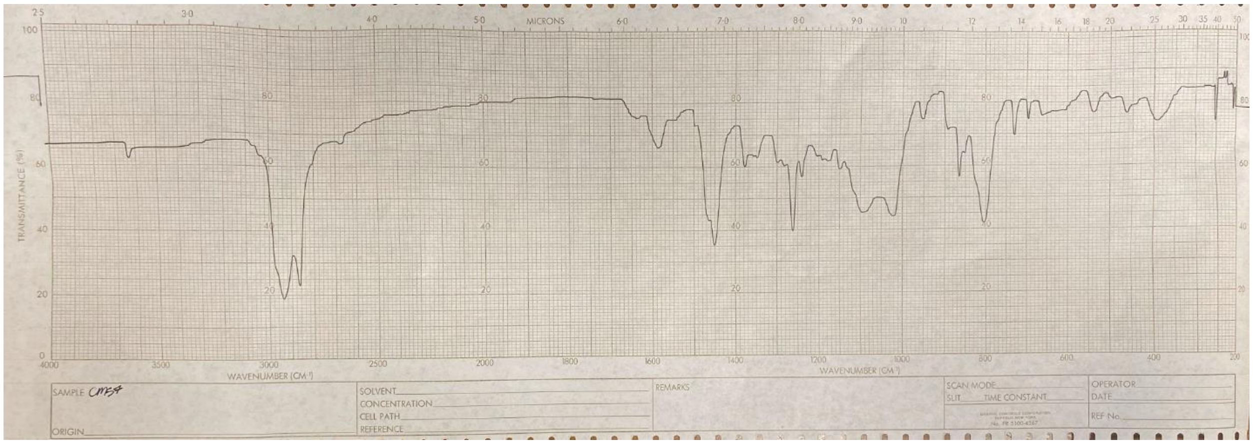

Figure S9. IR spectrum (Nujol) of $[Co(OC_6H_2\text{-}2,4,6\text{-}Cy_3)_2]_2$ (**3**).

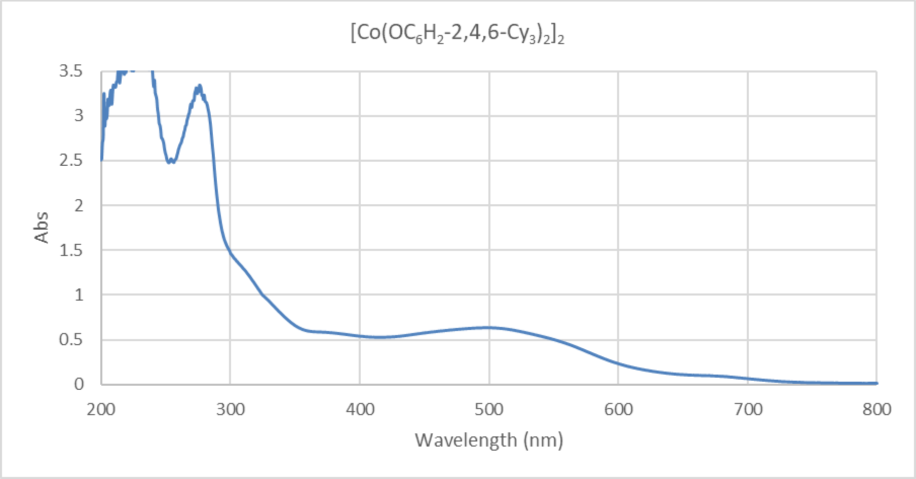

Figure S10. UV-Vis spectrum (430 µM, Hexanes) of $[Co(OC_6H_2\text{-}2,4,6\text{-}Cy_3)_2]_2$ (**3**).

Table S1. Crystal Data and Structure Refinement for **1-4**

	**1**	**2**	**3**	**4**
Empirical formula	C114 H157 Mn2 O4	C114 H158 Fe2 O4	C110 H156 Co2 O4	C96 H138 Co2 O6
Formula weight	1701.27	1704.11	1660.22	1505.92
Temperature	190(2) K	190(2) K	129(2) K	190(2) K
Wavelength	1.54178 Å	1.54178 Å	0.71073 Å	1.54178 Å
Crystal system	Monoclinic	Monoclinic	Monoclinic	Triclinic
Space group	$P2_1/c$	$P2_1/c$	Pn	P-1
Unit Cell Dimensions	a = 20.4551(4) Å	a = 22.2390(4) Å	a = 11.1831(11) Å	a = 14.0455(3) Å
	b = 16.4037(3) Å	b = 16.4520(3) Å	b = 15.4573(16) Å	b = 14.9075(3) Å
	c = 29.6358(5) Å	c = 29.1451(5) Å	c = 27.205(3) Å	c = 15.7600(3) Å
	α= 90°.	α= 90°.	α= 90°.	α= 117.5510(10)°.
	β= 91.7117(12)°.	β= 110.9159(10)°.	β= 93.546(3)°.	β= 99.8410(10)°.
	γ = 90°.	γ = 90°.	γ = 90°.	γ = 92.2350(10)°.

Volume	9939.5(3) Å3	9960.8(3) Å3	4693.6(8) Å3	2856.18(10) Å3
Z	4	4	2	1
Density (calculated)	1.137 Mg/m3	1.136 Mg/m3	1.175 Mg/m3	0.876 Mg/m3
Absorption coefficient	2.445 mm-1	2.713 mm-1	0.405 mm-1	2.568 mm-1
F(000)	3692	3704	1804	816
Crystal size	0.251 x 0.160 x 0.110 mm3	0.231 x 0.219 x 0.197 mm3	0.530 x 0.416 x 0.354 mm3	0.220 x 0.208 x 0.184 mm3
Crystal color and habit	Green Rectangular	Yellow Rectangular	Red Rectangular	Dark Red Cubic
Diffractometer	Bruker Photon2 CMOS	Bruker Photon2 CMOS	Bruker Photon2 CMOS	Bruker Photon100 CMOS
Theta range for data collection	2.161 to 68.678°.	2.127 to 72.718°.	2.251 to 25.756°.	3.223 to 68.665°.
Index ranges	-24<=h<=24, -19<=k<=19, -32<=l<=35	-27<=h<=27, -20<=k<=20, -36<=l<=36	-13<=h<=13, -18<=k<=18, -32<=l<=33	-16<=h<=16, -18<=k<=18, -19<=l<=19
Reflections collected	62245	69955	21594	19458
Independent reflections	18345 [R(int) = 0.0265]	19726 [R(int) = 0.0259]	21594 [R(int) = ?]	10480 [R(int) = 0.0162]
Observed reflections (I > 2sigma(I))	15702	15557	19667	9306
Completeness to theta = 67.679°	100.00%	100.00%	92.80%	99.70%
Absorption correction	Semi-empirical from equivalents	Semi-empirical from equivalents	Semi-empirical from equivalents	Semi-empirical from equivalents
Max. and min. transmission	0.6692 and 0.5844	0.6689 and 0.5499	0.8710 and 0.6999	0.7531 and 0.6263
Solution method	SHELXT (Sheldrick, 2015) Acta Cryst., A71, 3-8	SHELXT (Sheldrick, 2015) Acta Cryst., A71, 3-8	SHELXS-97 (Sheldrick, 1997)	SHELXS-97 (Sheldrick, 1997)
Refinement method	SHELXL-2018/3 (Sheldrick, 2018) Full-matrix least-squares on F2	SHELXL-2018/3 (Sheldrick, 2018) Full-matrix least-squares on F2	SHELXL-2018/3 (Sheldrick, 2018) Full-matrix least-squares on F2	SHELXL-2018/3 (Sheldrick, 2018) Full-matrix least-squares on F2
Data / restraints / parameters	18345 / 1632 / 1750	19726 / 909 / 1223	21594 / 94 / 1024	10480 / 708 / 595
Goodness-of-fit on F2	1.085	1.079	1.018	1.085
Final R indices [I>2sigma(I)]	R1 = 0.0540, wR2 = 0.1535	R1 = 0.0966, wR2 = 0.2407	R1 = 0.0667, wR2 = 0.1765	R1 = 0.0656, wR2 = 0.1964
R indices (all data)	R1 = 0.0610, wR2 = 0.1615	R1 = 0.1091, wR2 = 0.2493	R1 = 0.0739, wR2 = 0.1828	R1 = 0.0703, wR2 = 0.2018
Largest diff. peak and hole	0.745 and -0.375 e.Å-3	1.064 and -0.510 e.Å-3	0.522 and -0.519 e.Å-3	0.707 and -0.557 e.Å-3

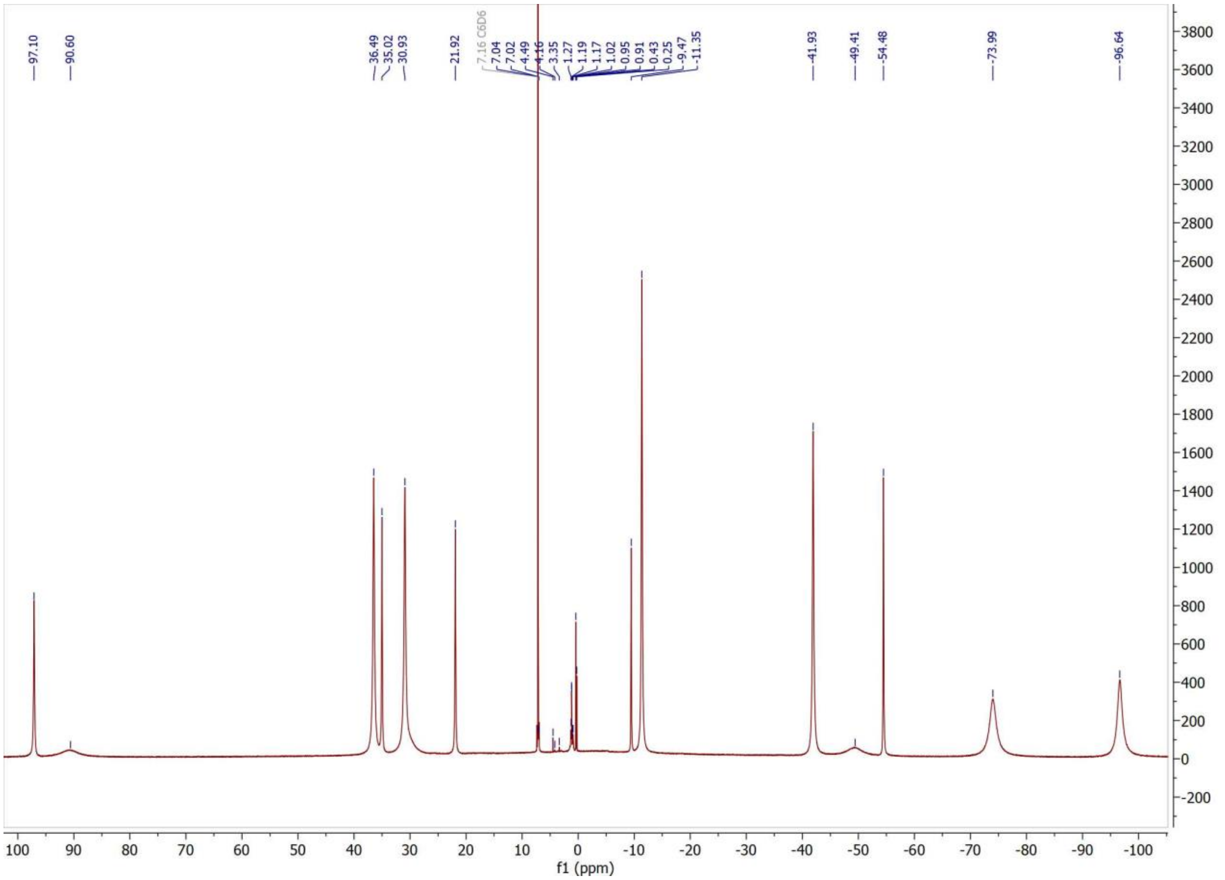

Figure S11. ^{1}H-NMR spectrum (C_6D_6, 400 MHz, 25 °C) of $[Fe(OC_6H_3\text{-}2,6\text{-}Pr^i_2)_2]_3$ (**5**).

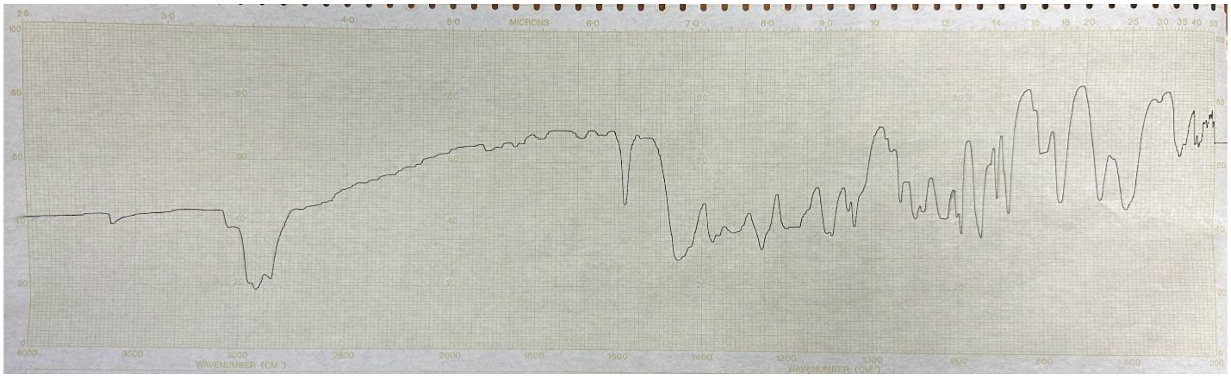

Figure S12. IR spectrum (Nujol) of $[Fe(OC_6H_3\text{-}2,6\text{-}Pr^i_2)_2]_3$ (**5**).

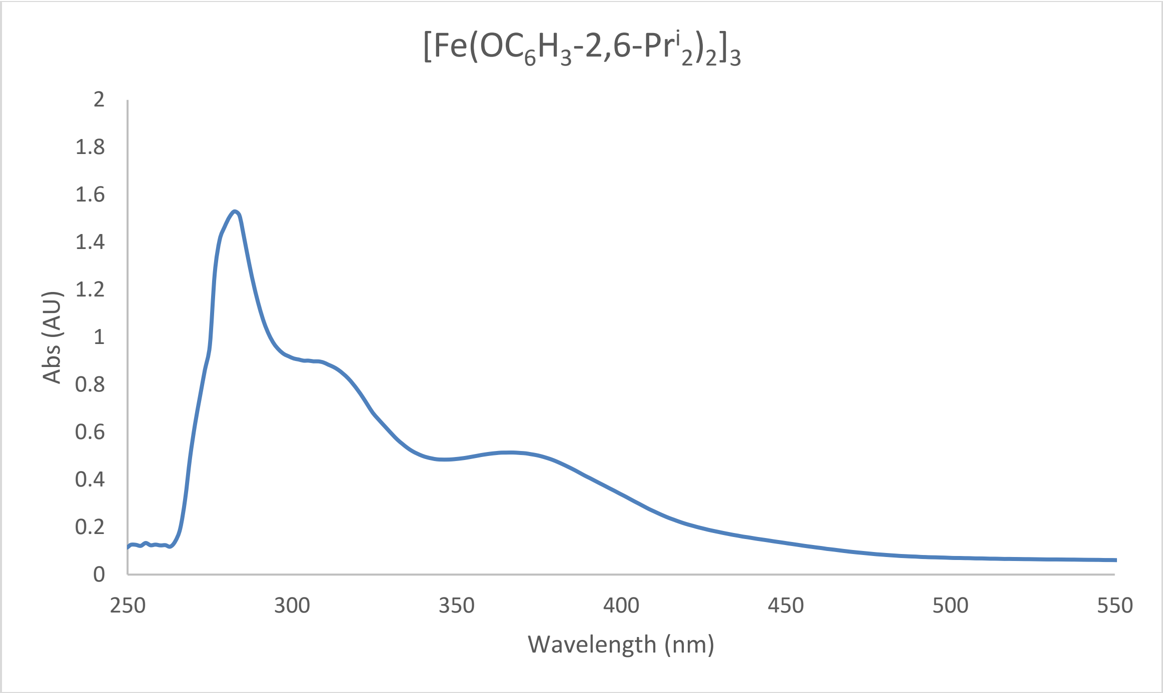

Figure S13. UV-Vis spectrum (89 µM, Toluene) of $[Fe(OC_6H_3\text{-}2,6\text{-}Pr^i{}_2)_2]_3$ (**5**).

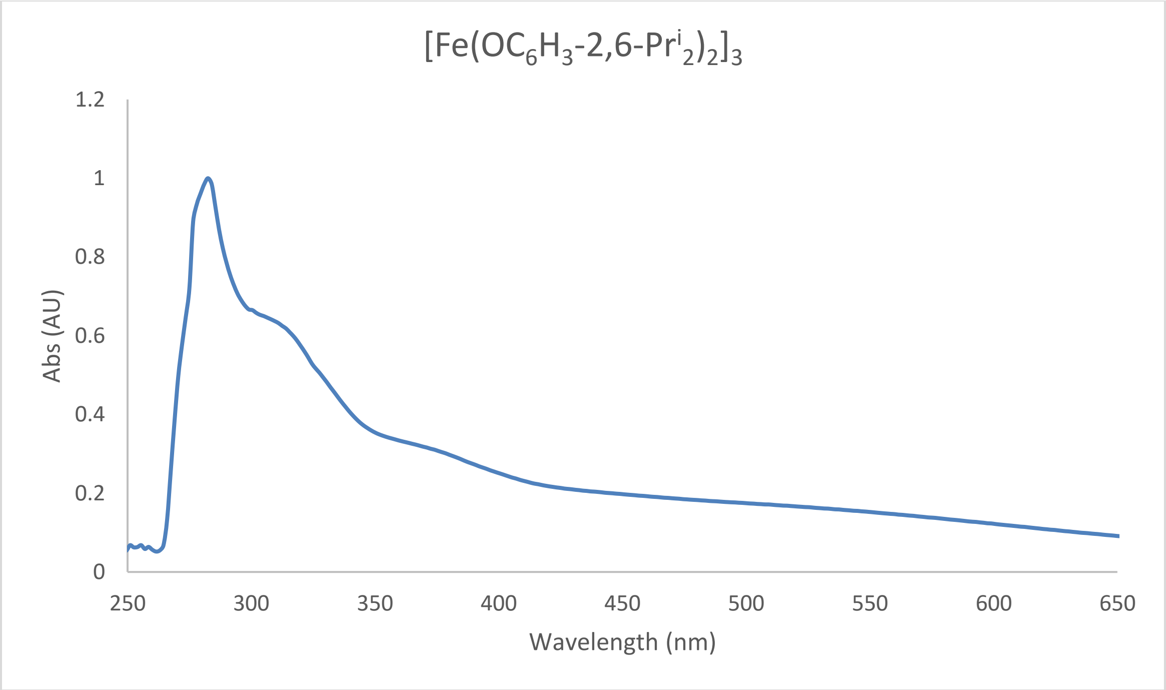

Figure S14. UV-Vis spectrum (45 µM, Toluene) of $[Fe(OC_6H_3\text{-}2,6\text{-}Pr^i{}_2)_2]_3$ (**5**).

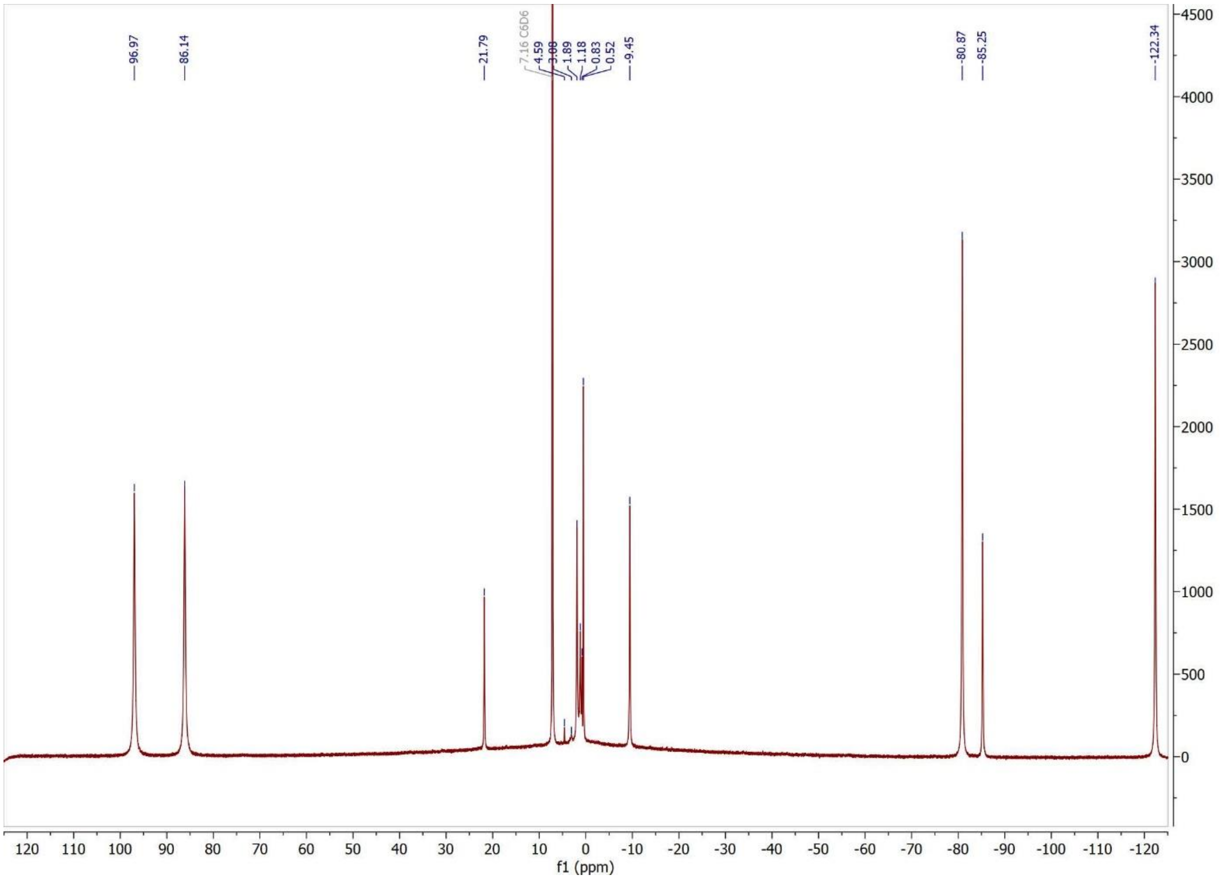

Figure S15. ^{1}H-NMR spectrum (C_6D_6, 400 MHz, 25 ^{o}C) of [Co(OC_6H_3-2,6-Pr^{i}_2)$_2$]$_3$ (**6**).

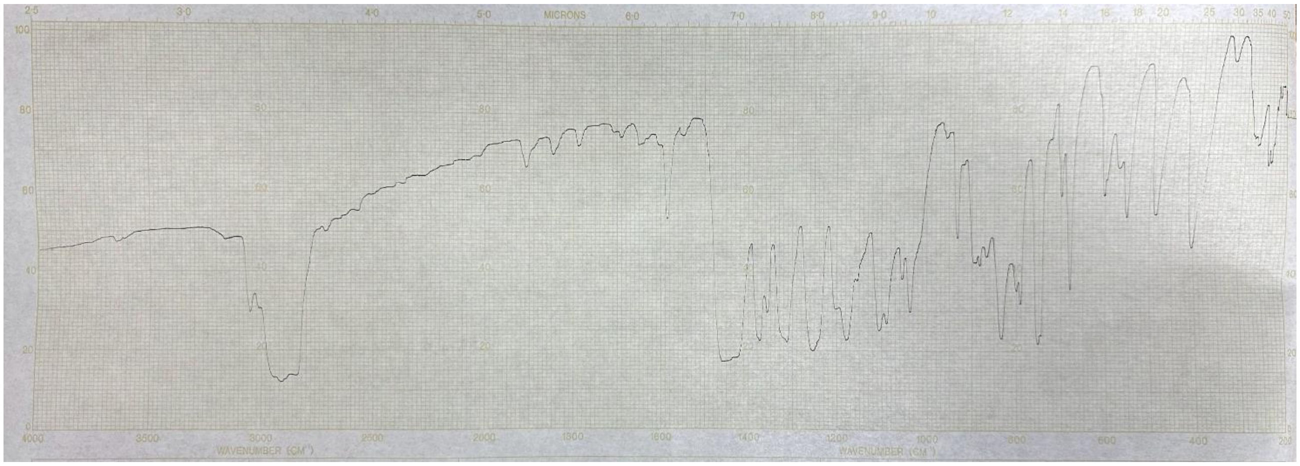

Figure S16. IR spectrum (Nujol) of [Co(OC_6H_3-2,6-Pr^{i}_2)$_2$]$_3$ (**6**).

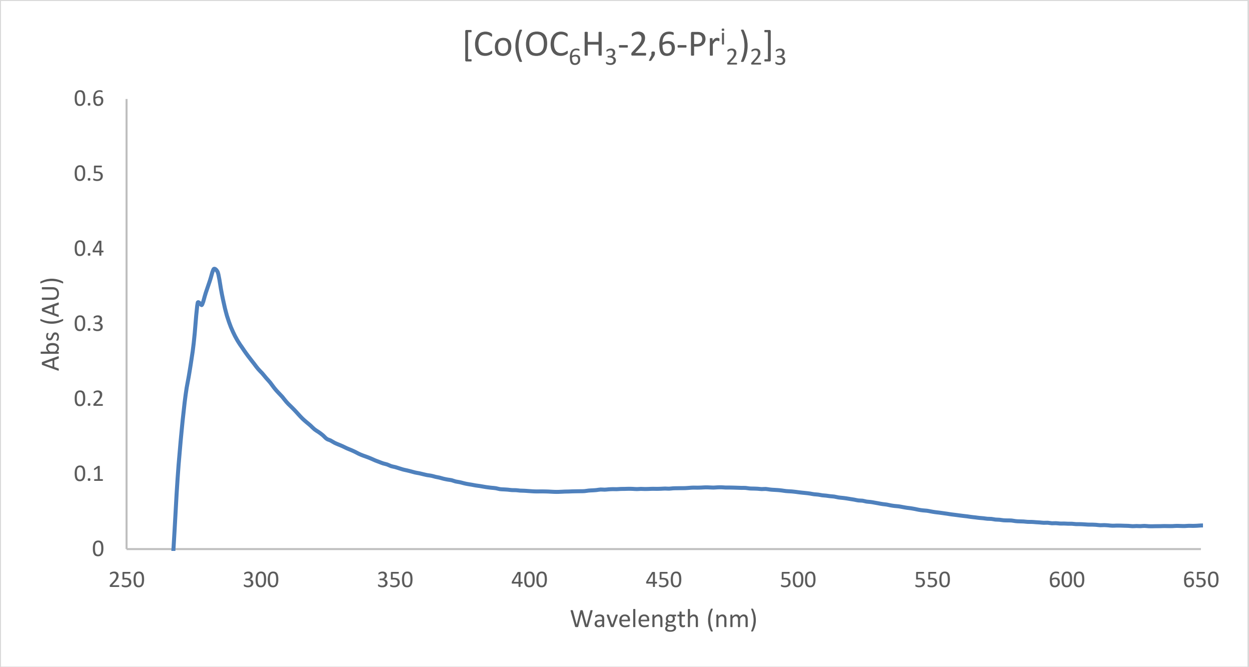

Figure S17. UV-Vis spectrum (27 µM, Toluene) of $[Co(OC_6H_3\text{-}2,6\text{-}Pr^i{}_2)_2]_3$ (**6**).

Table S2. Crystal Data and Structure Refinement for **5-6**.

	**5**	**6**
Empirical formula	C90 H120 Fe3 O6	C72 H102 Co3 O6
Formula weight	1465.4	1240.32
Temperature	90(2) K	190(2) K
Wavelength	0.71073 Å	0.71073 Å
Crystal system	Monoclinic	Monoclinic
Space group	$P2_1/c$	$P2_1/n$
Unit Cell Dimensions	a = 31.409(2) Å	a = 12.8237(4) Å
	b = 15.8667(12) Å	b = 22.2669(8) Å
	c = 35.229(3) Å	c = 24.3950(8) Å
	α= 90°.	α= 90°.
	β= 111.404(4)°.	β= 96.4980(12)°.
	γ = 90°.	γ = 90°
Volume	16345(2) $Å^3$	6921.1(4) $Å^3$
Z	8	4
Density (calculated)	1.191 Mg/m^3	1.190 Mg/m^3
Absorption coefficient	0.576 mm^{-1}	0.758 mm^{-1}
F(000)	6288	2652
Crystal size	0.359 x 0.256 x 0.188 mm^3	0.852 x 0.241 x 0.112 mm^3
Crystal color and habit	Green Rectangular Block	Red Rectangular
Diffractometer	Bruker APEX-II CCD	Bruker Photon2 CMOS

Theta range for data collection	0.696 to 27.703°.	2.098 to 27.531°.
Index ranges	-40<=h<=40, -20<=k<=20, -46<=l<=45	-11<=h<=16, -28<=k<=26, -31<=l<=31
Reflections collected	144864	44557
Independent reflections	38091 [R(int) = 0.0605]	15888 [R(int) = 0.0246]
Observed reflections (I > 2sigma(I))	24977	12229
Completeness to theta = 25.242°	100.00%	99.70%
Absorption correction	Semi-empirical from equivalents	Semi-empirical from equivalents
Max. and min. transmission	0.7536 and 0.6847	0.7979 and 0.6822
Solution method	SHELXT (Sheldrick, 2015) Acta Cryst., A71, 3-8	SHELXT (Sheldrick, 2015) Acta Cryst., A71, 3-8
Refinement method	SHELXL-2018/3 (Sheldrick, 2018) Full-matrix least-squares on F^2	SHELXL-2018/3 (Sheldrick, 2018) Full-matrix least-squares on F^2
Data / restraints / parameters	38091 / 130 / 1846	15888 / 0 / 754
Goodness-of-fit on F^2	1.04	1.039
Final R indices [I>2sigma(I)]	R1 = 0.0474, wR2 = 0.1138	R1 = 0.0356, wR2 = 0.0894
R indices (all data)	R1 = 0.0849, wR2 = 0.1338	R1 = 0.0520, wR2 = 0.0966
Largest diff. peak and hole	0.851 and -0.886 e.Å^{-3}	0.481 and -0.479 e.Å^{-3}
